# Disruption of the bacterial OLE RNP complex impairs growth on alternative carbon sources

**DOI:** 10.1093/pnasnexus/pgae075

**Published:** 2024-02-12

**Authors:** Seth E Lyon, Freya D R Wencker, Chrishan M Fernando, Kimberly A Harris, Ronald R Breaker

**Affiliations:** Department of Molecular Biophysics and Biochemistry, Yale University, New Haven, CT 06511, USA; Howard Hughes Medical Institute, Yale University, New Haven, CT 06511, USA; Department of Molecular Biophysics and Biochemistry, Yale University, New Haven, CT 06511, USA; Department of Molecular, Cellular and Developmental Biology, Yale University, New Haven, CT 06511, USA; Department of Molecular Biophysics and Biochemistry, Yale University, New Haven, CT 06511, USA; Howard Hughes Medical Institute, Yale University, New Haven, CT 06511, USA; Department of Molecular, Cellular and Developmental Biology, Yale University, New Haven, CT 06511, USA

**Keywords:** bTOR, noncoding RNA, protein secretion, Mn^2+^, *ykoY*

## Abstract

Ornate, large, extremophilic (OLE) RNAs comprise a class of large noncoding RNAs in bacteria whose members form a membrane-associated ribonucleoprotein (RNP) complex. This complex facilitates cellular adaptation to diverse stresses such as exposure to cold, short-chain alcohols, and elevated Mg^2+^ concentrations. Here, we report additional phenotypes exhibited by *Halalkalibacterium halodurans* (formerly called *Bacillus halodurans*) strains lacking functional OLE RNP complexes. Genetic disruption of the complex causes restricted growth compared to wild-type cells when cultured in minimal media (MM) wherein glucose is replaced with alternative carbon/energy sources. Genetic suppressor selections conducted in glutamate MM yielded isolates that carry mutations in or near genes relevant to Mn^2+^ homeostasis (*ykoY* and *mntB*), phosphate homeostasis (*phoR*), and putative multidrug resistance (*bmrCD*). These functional links between OLE RNA, carbon/energy management, and other fundamental processes including protein secretion are consistent with the hypothesis that the OLE RNP complex is a major contributor to cellular adaptation to unfavorable growth conditions.

**Significance Statement**

Ornate, large, extremophilic (OLE) RNAs represent one of the most complex and well-conserved classes of bacterial large noncoding RNAs discovered to date, yet their exact biological functions remain ambiguous. In the haloalkaliphilic bacterium, *Halalkalibacterium halodurans*, the ∼600-nt OLE RNA is highly abundant in the cell and its disruption causes growth defects under diverse stress conditions. In this study, we report that the absence of the OLE ribonucleoprotein complex hampers bacterial growth in minimal media with alternative carbon sources. This growth restriction phenotype is correlated with an impairment in protein secretion and can be overcome by supplementation with Mn^2+^ or by suppressor mutations associated with Mn^2+^ homeostasis. These findings further expand the scope of cellular processes associated with this large bacterial noncoding RNA.

## Introduction

Large noncoding RNAs (ncRNAs) are rare in bacteria but typically perform ancient and widely essential biological functions. The most prevalent classes are highly conserved across all (or nearly all) bacteria, which include the 23S and 16S ribosomal RNAs (1), RNase P ribozymes (2), transfer-messenger RNAs (3, 4), signal recognition particle RNAs (5), and self-splicing ribozymes (6, 7). Although additional classes of bacterial large ncRNAs have been reported (8), the identification of their biochemical and biological functions can be hindered by many factors, including their prevailing occurrence in organisms that are unculturable and/or are not conducive to genetic manipulation (9). However, the study of these RNAs offers opportunities to uncover biochemical mechanisms relevant to fundamental biological processes. Some of these large ncRNAs might even represent RNA devices that date back to the RNA World—a time before the evolutionary emergence of proteins (10, 11).

Notable among these mysterious classes of large ncRNAs is ornate, large, extremophilic (OLE) RNA, a ∼600-nt RNA mostly found in extremophilic and/or anaerobic species of Bacillota. OLE RNAs have been implicated in various bacterial stress responses, although the exact biochemical functions of this highly structured ncRNA remain elusive. The salt- and alkali-tolerant bacterium *Halalkalibacterium halodurans*, formerly known as *Bacillus halodurans* (12), is a genetically tractable model organism (13) that naturally carries an *ole* gene (14). In this species, OLE RNA is known to form a ribonucleoprotein (RNP) complex with at least three different proteins that are essential for its function: OapA (15), OapB (16–18), and OapC (19). The interaction of OapA with OLE RNA is required to localize the OLE RNP complex to the bacterial membrane (15). The small proteins OapB and OapC are members of distinct RNA binding protein classes (20, 21) that likely function as OLE RNA folding chaperones (16–19). All three proteins are necessary for *H. halodurans* to adapt to diverse stress conditions such as cold (≤20 °C) (16), the presence of short-chain alcohols [e.g. ethanol (∼5% v/v)] (22), or the presence of modestly elevated levels of Mg^2+^ (>4 mM) (23).

Given these diverse phenotypes and other observations, we recently hypothesized (24) that the OLE RNP complex is the bacterial functional equivalent of mTOR (mammalian target of rapamycin) complexes of eukaryotic cells (25, 26). Eukaryotes use mTOR complexes to coordinate responses to diverse stresses by regulating numerous metabolic and physiologic adaptations (25, 26). If OLE RNA does participate in the formation of a “bTOR” (bacterial tasks OLE regulates) device that serves purposes like those of mTOR, then we predict that many other functions of OLE RNP complexes remain to be discovered. Furthermore, we have proposed (24) that similar regulatory networks exist in bacteria that lack the *ole* gene. Therefore, investigations into the functions of OLE RNP complexes may provide insight into stress response mechanisms utilized by many bacterial species.

In the current study, we investigated the possibility that OLE RNP complexes are involved in cellular adaptation to suboptimal carbon sources when glucose is not available. This effort was motivated by the fact that several links between OLE RNA and carbon metabolism pathways have been identified in previous studies. For example, an OLE RNA pull-down analysis (19) revealed that the RNA interacts with the ArgR protein, a transcriptional regulator of arginine metabolism and carbon metabolism pathways (27, 28). Furthermore, the *argR* gene often resides near the *ole* and *oapA* genes (16), which supports the conclusion that there are both physical and functional links between the ArgR protein and OLE RNA.

To search for additional links between OLE RNP complexes and carbon/energy management, we assessed the ability of *H. halodurans* strains lacking genes for both OLE RNA and OapA protein (Δ*ole-oapA*) to grow on various gluconeogenic carbon sources, including TCA (tricarboxylic acid) cycle intermediates, amino acids, and pyruvate. Functional OLE RNP complexes were found to be necessary for robust growth on nearly any alternative carbon source tested, with glutamate causing the greatest growth disparity in Δ*ole-oapA* cells. Genetic suppressor selections using the Δ*ole-oapA* strain revealed that the majority of mutations that restore robust growth with suboptimal carbon sources were located in a Mn^2+^-sensing riboswitch (29–31) associated with the *ykoY* gene (recently (32) renamed to *meeY*), or directly in the *ykoY* open reading frame (ORF), which encodes a protein involved in facilitating protein secretion and exoprotein metalation (32, 33).

Furthermore, the Δ*ole-oapA* strain exhibits reduced protein secretion compared to the wild-type (WT) strain in milk agar secretion assays and grows nearly 100-fold worse than WT when using intact casein protein as the carbon source in minimal media (MM). Casein requires digestion by secreted, extracellular proteases prior to the import of proteolysis products as oligopeptides and amino acids to be used as carbon sources. These findings implicate OLE RNA as an important participant in the utilization of nonpreferred carbon sources, and also in protein secretion, which expands the fundamental cellular processes linked to the function of this unusual bacterial ncRNA.

## Results and discussion

### Evidence that the OLE RNP complex is relevant to carbon/energy metabolism

Previous studies provide indications that the function of the OLE RNP complex is important for growth on nonpreferred carbon sources. For example, the gene for OLE RNA often resides near genes whose protein products are relevant to carbon (*folD*, *argR*) and energy (*nadF*) management (14, 16, 24). Genes are often clustered in bacterial genomes when they are involved in the same biological or biochemical processes (34). Indeed, the ArgR protein (arginine repressor, also called AhrC) interacts with OLE RNA inside *H. halodurans* cells (19), and this binding interaction has been confirmed by in vitro assays with recombinant ArgR protein (unpublished observations). ArgR is an arginine-responsive transcription factor that was originally characterized as a regulator of arginine metabolism genes (27, 28). However, a recent study in *Bacillus subtilis* demonstrates that the ArgR regulon is broader and that it enables cells to utilize glucose via regulation of Mn^2+^-homeostasis genes (35).

Furthermore, *argR* mRNA translation is known to be inhibited by the SR1 RNA (36), which is a small RNA whose expression is repressed by the transcriptional repressor protein CcpN when glucose levels are high (37). Thus, the expression of *argR* is regulated in part by the relative abundance of glucose, which is the preferred carbon/energy source of these bacteria. SR1 RNA also codes for a small peptide called SR1P that forms a biologically relevant complex with glyceraldehyde-3-phosphate dehydrogenase (GapA) (38), which catalyzes the sixth step in glycolysis. Intriguingly, the substrate of GapA, glyceraldehyde-3-phosphate, is also a substrate for the Dxs protein (1-deoxy-D-xylulose-5-phosphate synthase), an enzyme that catalyzes the first step in the formation of isoprenoid precursors (39) and whose gene often resides immediately adjacent to *ole* and *oapA* (14, 16, 24). Taken together, these observations suggest that there could be multiple physical and functional relationships between OLE RNA, other products of the *ole* gene cluster, and carbon/energy metabolism.

### The OLE RNP complex is required for robust growth on alternative carbon sources

**Fig. 1.**
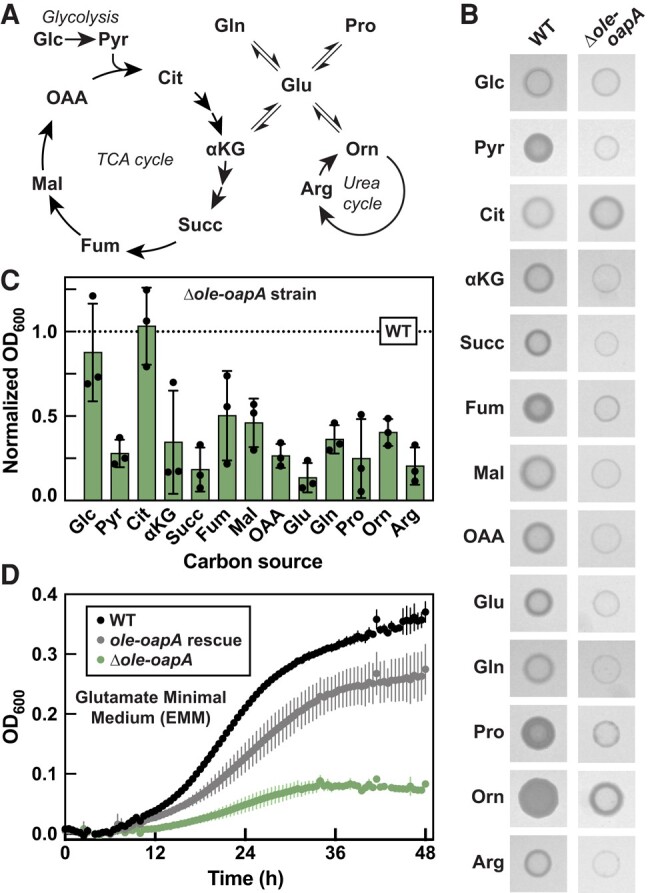
Disruption of the OLE RNP complex diminishes growth of *H. halodurans* on alternative carbon sources. A) Abbreviated schematic of central metabolism depicting metabolites tested as carbon sources in MM. B) Representative growth assays of *H. halodurans* WT and Δ*ole-oapA* strains on MM agar with the carbon sources as indicated in (A). Images were recorded after 40 h of growth at 37 °C. C) Growth assays of *H. halodurans* in MM with various carbon sources as indicated in (A). Bars represent the average OD_600_ (optical density at 600 nm) of Δ*ole-oapA* cultures normalized to the OD_600_ of WT (dashed line) when grown in MM having the same carbon source. This study was performed with three biological replicates that each were comprised of three technical replicates, wherein OD_600_ values (see Fig. S1) were recorded 48 h after inoculation. Error bars depict standard deviation. D) Representative growth curve assays of *H. halodurans* WT, Δ*ole-oapA* containing the pHCMC05 *ole-oapA* rescue vector, and Δ*ole-oapA* in glutamate minimal media (EMM). Data points represent averages from three technical replicates and error bars depict standard deviation. Glc, glucose; Pyr, pyruvate; Cit, citrate; αKG, α-ketoglutarate; Succ, succinate; Fum, fumarate; Mal, malate; OAA, oxaloacetate; Glu, glutamate; Gln, glutamine; Pro, proline; Orn, ornithine; Arg, arginine.

The role of the OLE RNP complex on bacterial growth using various carbon sources was evaluated by culturing WT and Δ*ole*-*oapA* strains of *H. halodurans* in MM supplemented with glucose or various gluconeogenic carbon sources (Fig. 1A). Importantly, we used a minimal medium (40) with scarce amounts of trace metals, which we expected would place a high metabolic burden on the bacterium by forcing it to synthesize its required metabolites de novo. MM containing different carbon sources supports the growth of WT *H. halodurans* cells to different extents (Figs. 1B and S1), suggesting that the bacteria are being challenged under these conditions. Most importantly, the Δ*ole-oapA* strain experienced severe growth impairments compared to WT cells on all carbon sources tested except for glucose and citrate, which enabled growth like that of WT (Figs. 1B and C and S1).

The observation that citrate as a sole carbon/energy source supports robust growth of Δ*ole*-*oapA* cells is notable because supplementation of citrate was previously (23) shown to overcome the growth deficiency caused by exposure of Δ*ole*-*oapA* cells to excess (>4 mM) Mg^2+^ ions. Furthermore, mutations that overcome the growth deficiency caused by excess Mg^2+^ occur in genes relevant to the TCA cycle, which presumably increases intracellular citrate levels. It has been proposed (23) that citrate might overcome Mg^2+^ toxicity due to its ability to chelate divalent metal ions. The relevance of citrate in both the carbon source and Mg^2+^ toxicity phenotypes could be due to a mechanistic link between the two phenotypes. However, the Mg^2+^ concentration used in the carbon source experiments is ∼0.8 mM, and therefore it is unlikely that Δ*ole-oapA* cells are experiencing Mg^2+^ stress in the present growth assays. Although the possible connection between citrate and the carbon source and Mg^2+^ stress phenotypes merits further investigation, in the current study, we chose to focus our analyses on carbon sources where Δ*ole-oapA* cells failed to grow to WT levels.

Minimal medium supplemented with glutamate as the carbon/energy source, called glutamate minimal medium (abbreviated EMM), was chosen for subsequent experiments because it provided the largest growth difference between WT and Δ*ole*-*oapA* strains (Figs. 1B–D and S1). In addition to its role in protein biosynthesis, glutamate is positioned at the intersection of several metabolic pathways (Fig. 1A) and serves various other purposes ranging from supplying nitrogen for several biochemical reactions to contributing to cell wall production (e.g. polyglutamate), among many other roles (41). Importantly, the diminished growth phenotype of Δ*ole*-*oapA* cells on EMM can be largely overcome by expressing *ole* and *oapA* genes from a plasmid (Fig. 1D). Individual Δ*ole* and Δ*oapA* strains of *H. halodurans* also exhibit similar growth deficiencies in EMM (Fig. S2), which is consistent with the conclusion that robust growth in EMM depends on an intact OLE RNP complex.

### Suppressor selections in EMM often yield mutations in genes relevant to Mn^2+^

After prolonged incubation in liquid EMM, all strains of *H. halodurans* with disrupted OLE RNP complexes (Δ*ole*-*oapA*, Δ*ole*, or Δ*oapA*) eventually reach cell densities comparable to WT cultures. We speculated that suppressor mutations were occurring that permitted robust growth despite the absence of a functional OLE RNP complex. As expected, whole-genome sequencing (WGS) confirmed that suppressor mutations were occurring.

**Fig. 2.**
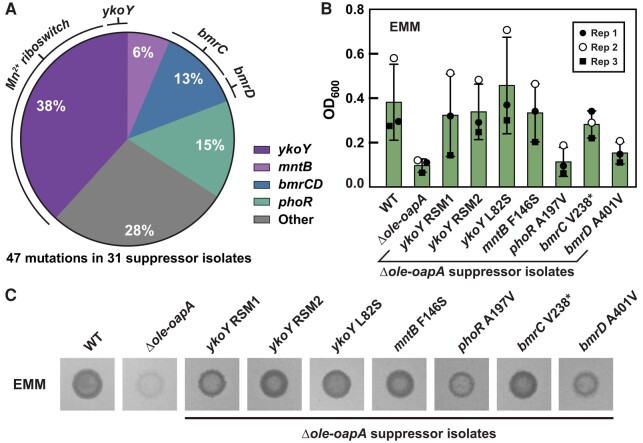
*Halalkalibacterium halodurans* Δ*ole-oapA* strain spontaneously acquires suppressor mutations that restore robust growth on EMM. A) Depiction of the distribution of mutations (47 in total) observed across 31 unique *H. halodurans* Δ*ole-oapA* EMM suppressor isolates. Silent mutations were excluded. The Mn^2+^ riboswitch is located immediately upstream of the *ykoY* ORF. The protein products encoded by *bmrC* and *bmrD* form a heterodimer. B) Growth assays in liquid EMM with *H. halodurans* WT, Δ*ole-oapA*, and Δ*ole-oapA* carrying various suppressor mutations as indicated. Biological replicates (*n* = 3) were performed on separate days and are indicated as Rep 1 through Rep 3. Bars indicate average OD_600_ values from biological replicates wherein each replicate was comprised of three technical replicates. Error bars indicate standard deviation. C) Representative growth assay on EMM agar with various suppressor isolates after 4 days of growth at 37 °C. Agar spot assays were performed with at least three independent biological replicates and each biological replicate was spotted on EMM agar three separate times, wherein all gave results similar to those depicted.

A total of 47 mutations were identified among 31 Δ*ole-oapA* suppressor isolates subjected to WGS that exhibited evidence of bacterial growth after prolonged incubation in liquid EMM (Fig. 2A and Table S1). Similarly, a total of 37 mutations were identified among 27 Δ*ole* suppressor isolates (Table S2). Thus, several suppressor isolates experienced at least two distinct mutations, whereas others acquired only a single mutation. There is considerable overlap in the genes acquiring mutations from the Δ*ole-oapA* and Δ*ole* suppressor strains, indicating that the mutations in these genes likely are responsible for suppression of the minimal medium growth limitation phenotype.

Most mutations observed were found in genes related to divalent manganese (Mn^2+^) biology (Figs. 2A and S3). For both Δ*ole-oapA* and Δ*ole* strains, the most abundant suppressor mutation (Fig. 2A) is a deletion flanked upstream by a “*yybP-ykoY*” RNA motif (29) and downstream by the *ykoY* ORF. Representatives of the *yybP-ykoY* motif class of RNAs are known to function as Mn^2+^-responsive riboswitches (30, 31). These suppressor mutations are predicted to disrupt the intrinsic terminator stem (42, 43) of the riboswitch expression platform (44, 45). As discussed later, this common riboswitch mutation is predicted to increase *ykoY* expression and yield more YkoY protein. The YkoY protein is a predicted membrane protein that carries a TerC domain and was recently demonstrated to facilitate the Sec-dependent secretion of Mn^2+^-binding proteins (32).

Several additional mutations were identified in other genes associated with Mn^2+^ biology or homeostasis. Some suppressor isolates carry a missense mutation at a highly conserved leucine residue (L82S) encoded by the *ykoY* gene (Fig. S4). Other suppressor isolates carry unique missense mutations in the manganese homeostasis genes *mntB* and *mntC* that, respectively, encode the ATPase and permease components of the MntABC Mn^2+^ importer (46). These findings strongly implicate Mn^2+^ as a key factor in the carbon source growth phenotype caused by disruption of the OLE RNP complex.

The second most frequently mutated gene is *phoR*, which encodes a phosphate-sensing sensor histidine protein kinase (47). Only one unique missense mutation to this gene is observed at a poorly conserved position, which results in an alanine-to-valine substitution. The remaining mutations that occur more than once were found in *bmrC* and *bmrD*, whose protein products are known to form a heterodimer proposed to function as a multidrug efflux pump (48, 49). Several unique nonsense mutations occur in *bmrC* that result in protein truncation, whereas only missense mutations are observed in *bmrD*. Although little is known about the function of the BmrCD complex, the expression of these proteins is triggered by ribosome stalling via a transcription attenuation mechanism (50). In addition, *B. subtilis* BmrD is known to interact with KinA, a kinase that initiates a phosphorelay process to trigger sporulation (51). *Halalkalibacterium halodurans*, however, does not appear to have a close homolog of KinA and thus a sporulation role for BmrCD in this species is uncertain.

Lastly, two suppressor mutations emerged in the Δ*ole* strain that reside in the *icd* gene, which codes for isocitrate dehydrogenase (see also the discussion above regarding citrate). The occurrence of mutations in *icd*, *bmrC*, and *bmrD* in the carbon-source suppressor selections described herein are notable in part because mutations in these genes were previously found in independent genetic suppressor selections for *H. halodurans* Δ*ole-oapA* cells under Mg^2+^ stress (23). This overlap in genetic suppressor hits raises the possibility that similar disruptive mechanisms might be at play when bacteria lacking the OLE RNP complex are grown under carbon- or Mg^2+^-stress conditions.

### Individual suppressor mutations improve growth in EMM

To investigate the impact of the acquired suppressor mutations, the growth characteristics of certain suppressor isolates that carry only a single genetic alteration were evaluated. All Δ*ole-oapA* suppressor isolates carrying a single genetic change surpassed the growth of the *H. halodurans* Δ*ole-oapA* parent strain when grown in EMM in both liquid culture (Fig. 2B) and on solid agar (Fig. 2C). These results confirm that each individual genetic change identified in the current study provides a growth improvement to the Δ*ole-oapA* parent strain and suggest that the functions of the mutated components are relevant to the function of the OLE RNP complex.

Notably, the suppressor mutations facilitate growth in EMM to different extents. Suppressor isolates containing mutations in genes pertaining to Mn^2+^ biology [*ykoY* riboswitch mutant 1 (RSM1), *ykoY* RSM2, *ykoY* L82S, and *mntB* F146S] were observed to grow nearly as well as WT. Furthermore, the *bmrC* V238* suppressor isolate also permitted growth to near WT levels. However, despite being the second most frequent suppressor mutation, the *phoR* A197V change yields growth that is only modestly better than the parent Δ*ole-oapA* strain (Fig. 2B and C). Such partial suppression effects could explain why some suppressor isolates carry more than one mutation (see Tables S1 and S2) that most likely additively improve growth characteristics in EMM.

### Carbon-source suppressor mutations also overcome other Δ*ole*-associated phenotypes

**Fig. 3.**
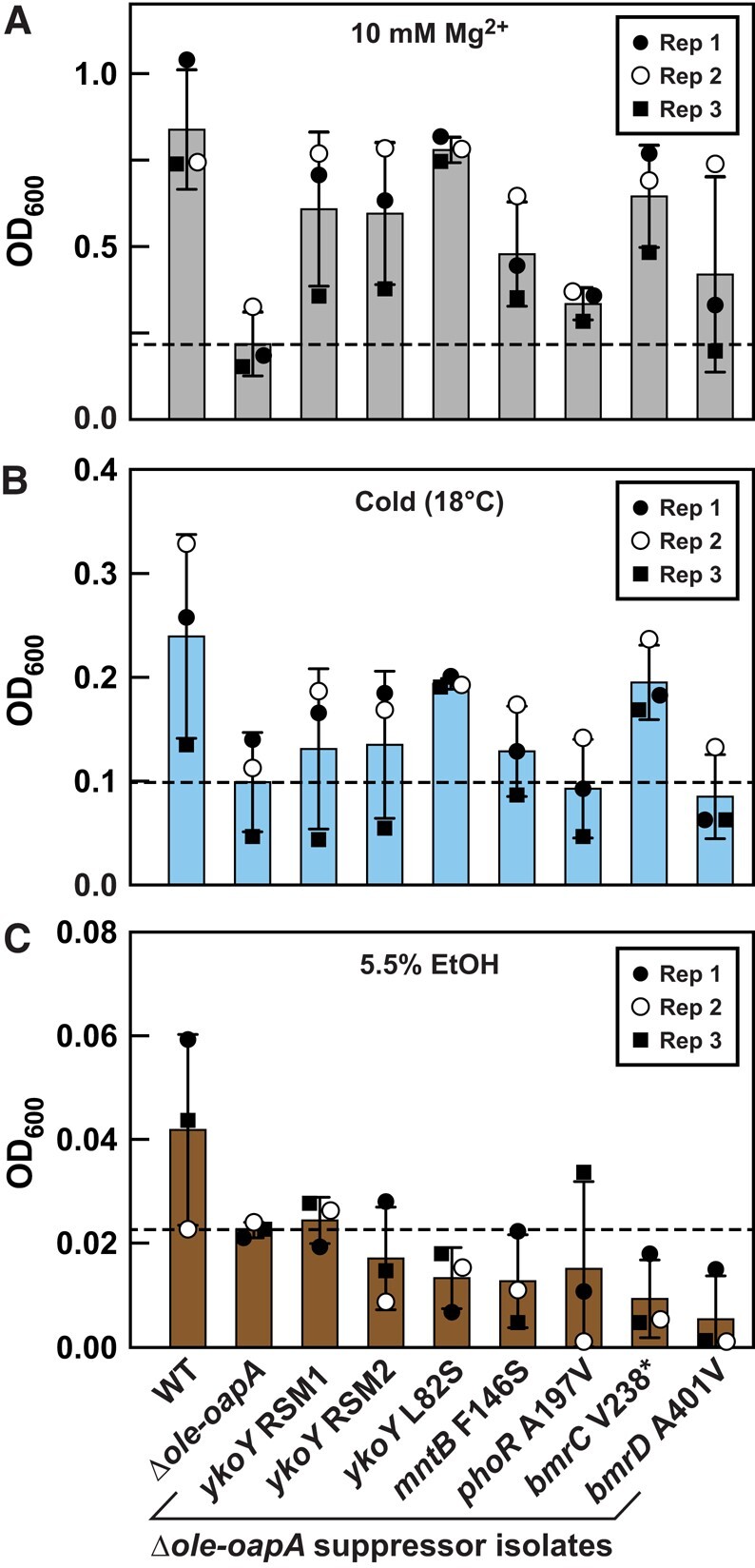
Some EMM suppressor mutants also confer resistance to Mg^2+^ and cold stresses, but not ethanol stress. A) Growth assays with various *H. halodurans* Δ*ole-oapA* EMM suppressor isolates (as annotated in C) cultured in LB (pH 10) supplemented with 10 mM Mg^2+^ at 37 °C for 48 h. B) Growth assays conducted in LB (pH 10) and grown at 18 °C for 72 h. C) Growth assays conducted in LB (pH 10) supplemented with ethanol [5.5% v/v] and grown at 37 °C for 48 h. Biological replicates (*n* = 3) from each experiment were performed on separate days and are indicated as Rep 1 through Rep 3. Bars indicate average OD_600_ from biological replicates wherein each replicate was comprised of three technical replicates. Error bars indicate standard deviation.

Two genetic loci that acquired mutations in the carbon-source suppressor selections, *icd* and *bmrC* (Fig. S3), also were identified as suppressors of Mg^2+^ toxicity (23). Thus, we evaluated other carbon-source suppressor isolates to determine if they could overcome other phenotypes observed when the OLE RNP complex is disrupted. Intriguingly, all carbon-source suppressor mutants tested also exhibit resistance to Mg^2+^ stress (Figs. 3A and S5, top) with improvements in growth like that observed when cultured in EMM. Similar results are observed for the same suppressor isolates when tested under cold stress (18 °C), except that the *phoR* and *bmrD* mutations did not improve growth under this condition (Figs. 3B and S5, bottom). These findings again suggest that some of the phenotypes caused by OLE RNP disruption are biochemically linked such that they can be overcome by the same suppressor mutations.

A different pattern was observed when the carbon-source suppressor isolates were tested for their ability to resist short-chain alcohol stress. None of the suppressor mutants overcame 5.5% [v/v] ethanol stress, but rather most appear to cause further growth impairment (Fig. 3C). Thus, although loss of the OLE RNP complex results in reduced growth in all four stresses, the carbon-source suppressor mutations identified cannot overcome all stresses. This suggests that multiple mechanisms might be involved in causing the observed phenotypes and that their disruption cannot be universally overcome by any single suppressor mutation.

### *ykoY* overexpression facilitates growth of Δ*ole-oapA* cells in EMM

The abundance of mutations in genes relevant to Mn^2+^ that suppress the carbon-source phenotype (Figs. 2A and S3) suggests that abnormally low intracellular Mn^2+^ concentrations might be one reason for the impaired growth of bacteria lacking a functional OLE RNP complex. It is known that Mn^2+^ concentrations must be maintained at sufficient levels to enable bacterial growth (52). For example, *B. subtilis* grows poorly on glucose when Mn^2+^ is limited, likely because the active sites of several glycolytic enzymes require Mn^2+^ for catalysis (53). We have previously noted (23, 24) that OapA, an essential OLE RNP complex partner, is most similar in sequence to Mg^2+^ transporters such as MpfA and YhdP. Based on these observations, we considered the possibility that the OLE RNP complex might influence the levels of Mg^2+^, Mn^2+^, or the ratio of these two metals, leading to the absence of Mn^2+^ at the active sites of certain enzymes.

**Fig. 4.**
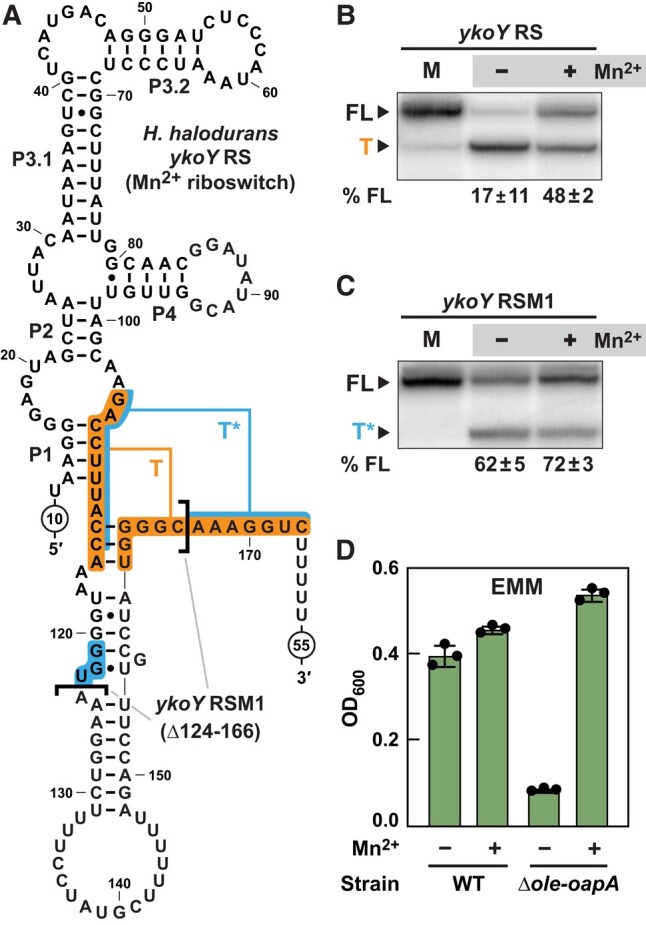
Δ*ole-oapA* suppressor isolates carrying a 43-base-pair deletion upstream of the *ykoY* gene disrupt a Mn^2+^ riboswitch to yield increased YkoY production. A) Sequence and secondary structure model for the *H. halodurans ykoY* riboswitch in its predicted Mn^2+^-bound state (genetic “ON” conformation). Brackets identify the 43-nucleotide RNA region deleted in the RSM1 mutation, which is a deletion of nucleotides 124 through 166. Indicated are the natural intrinsic terminator stem (T) and the predicted fortuitous terminator (T*) formed by the RSM1 suppressor mutant. B) Transcription termination assays using the WT *ykoY* riboswitch sequence (*ykoY* RS). FL, full-length transcript; T, terminated transcript at the T terminator; (‒), no supplemented Mn^2+^; (+), 200 µM supplemented MnCl_2_. % FL represents the average and standard deviation of FL transcripts based on three replicate experiments. C) Transcription termination assay using the *ykoY* RSM1 riboswitch identified in Δ*ole-oapA* suppressor selection in EMM. Annotations are as described for (B), except that T* designates the product band resulting from termination at the T* terminator. D) Growth assays in EMM with *H. halodurans* WT and Δ*ole-oapA* strains in the absence (−) or presence (+) of 10 µM MnCl_2_. Cells were grown for 48 h. Bars represent the average from three biological replicates wherein each replicate was comprised of three technical replicates. Error bars represent standard deviation.

As mentioned earlier, numerous suppressor isolates were identified that carried a 43-base-pair deletion upstream of the *ykoY* ORF. This recurring deletion appears to disrupt a critical intrinsic terminator hairpin (42, 43) of the expression platform for a Mn^2+^ riboswitch (30, 31) (Fig. 4A). Thus, we called this mutation *ykoY* riboswitch mutant 1 or “*ykoY* RSM1”. A similar deletion was also identified called *ykoY* RSM2, although we did not further investigate this suppressor mutant. The natural riboswitch sequence, herein termed *ykoY* RS, is predicted to function as a genetic “ON” switch that increases expression of the *ykoY* gene when Mn^2+^ is abundant. However, we hypothesized that the disruption of the terminator stem should result in an increase in YkoY production.

In vitro transcription termination assays were used to verify that the natural *ykoY* riboswitch is a Mn^2+^-responsive “ON” switch. In the absence of Mn^2+^, transcription strongly terminates at a site expected for the natural terminator (Fig. 4B and T). When 200 μM Mn^2+^ is present in the transcription reaction, nearly 50% of the transcripts read through the terminator to yield full-length (FL) RNAs (Fig. 4B and FL). Surprisingly, the *ykoY* RSM1 construct undergoes termination even though the natural terminator stem has been disrupted by the 43-nucleotide deletion (Fig. 4C). This appears to be caused by a second, fortuitous terminator stem that is formed from a portion of the original terminator sequence plus an additional three base-pairs (Fig. 4A, blue shading). Regardless, the change in the *ykoY* RSM1 RNA structure yields over 60% FL transcripts even in the absence of added Mn^2+^, which is an ∼3-fold increase in the percentage of FL transcript compared to the WT riboswitch.

In cells, the *ykoY* RSM1 mutation is expected to produce more FL *ykoY* mRNA and subsequently more YkoY protein. This hypothesis is supported by the fact that overexpression of the *ykoY* gene from a plasmid in *H. halodurans* Δ*ole-oapA* cells restores robust growth characteristics in EMM- and Mg^2+^-stress conditions (Fig. S6). Thus, bacteria lacking a functional OLE RNP complex might suffer from Mn^2+^ insufficiency, in part leading to insufficient YkoY levels when grown in EMM.

### Mn^2+^ supplementation facilitates growth of Δ*ole-oapA* cells in MM

To further evaluate the hypothesis that the disruption of the OLE RNP complex causes a deficiency of Mn^2+^, we assessed the effects of Mn^2+^ supplementation on the growth in EMM. As expected, the addition of Mn^2+^ to EMM greatly improves growth of the Δ*ole-oapA* strain but only modestly enhances the growth of WT (Figs. 4D and S7). Likewise, we confirmed that Mn^2+^ supplementation also rescues growth of the Δ*ole-oapA* strain to WT levels when cultured using various carbon sources (Fig. S8). These results suggest that Mn^2+^ deficiency is a major growth limitation of bacteria lacking a functional OLE RNP complex when grown in MM with most carbon sources other than glucose. Furthermore, we speculate that the *ykoY* and *mntBC* ORF mutations observed in other suppressor isolates are likely gain-of-function mutations that, respectively, increase YkoY activity and elevate intracellular Mn^2+^ levels.

Because some of the carbon-source suppressor mutants also exhibit improved growth in the presence of elevated Mg^2+^ levels and cold (Figs. 3A and B and S5), we sought to determine if Mn^2+^ supplementation also would improve the tolerance of Δ*ole-oapA* cells to elevated Mg^2+^, cold, or ethanol stresses. Growth assays reveal that excess Mn^2+^ supplementation improves growth of Δ*ole-oapA* cells when under Mg^2+^ stress but has no effect when cells experience cold or ethanol stress (Fig. S9). These results are consistent with our observations that carbon-source suppressor isolates carrying mutations expected to increase *ykoY* expression (*ykoY* RSM1*, ykoY* RSM2, and *mntB* F146S) also do not improve resistance to cold stress (Figs. 3B and S5). Neither does ectopic expression of *ykoY* from a plasmid (Fig. S6, right). These findings also suggest that the L82S mutation in YkoY provides a change in protein activity that cannot be fully recapitulated by simple overexpression of the WT protein.

### Disruption of the OLE RNP complex impairs protein secretion

As demonstrated above, either overexpression of YkoY protein or alteration of its amino acid sequence (L82S) overcomes the growth deficiency of the Δ*ole-oapA* strain under several stress conditions. As mentioned above, the YkoY (MeeY) protein has been suggested to serve a role in Mn^2+^ metalation of Sec-dependent secreted proteins, and YkoY deletion also contributes to the jamming of the Sec translocase machinery (32). These findings prompted us to hypothesize that protein secretion was impaired when *H. halodurans* Δ*ole-oapA* cells encounter stress. Initially, a qualitative assessment was conducted by growing bacteria on milk agar, which enables visual observation of the effects of secreted proteases that digest casein (54). Specifically, casein degradation results in a reduction of the white, opaque character of milk, which leads to an increase in transparency of the agar.

**Fig. 5.**
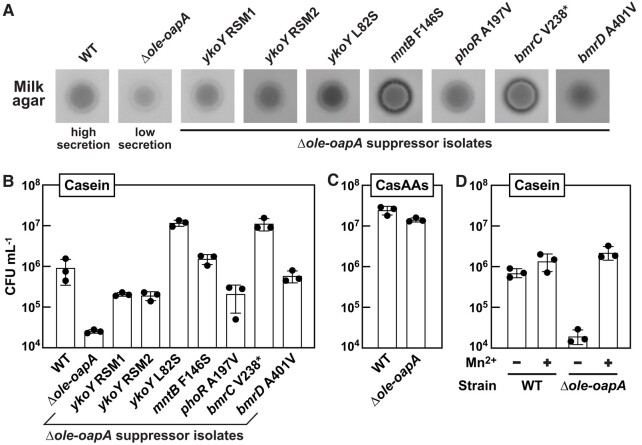
Growth of various *H. halodurans* Δ*ole-oapA* strains correlates with protein secretion when bacteria are grown on alternative carbon sources. A) Protein secretion assays of *H. halodurans* WT, Δ*ole-oapA*, and Δ*ole-oapA* suppressor isolates as indicated using milk agar plates. Darker agar zones indicate a greater level of secretion of proteases by the bacteria. B) Quantification of CFU with *H. halodurans* WT, Δ*ole-oapA*, and Δ*ole-oapA* suppressor isolates cultured after 40 h incubation in MM using casein [1% w/v] as the carbon source. Casein requires degradation by secreted proteases prior to utilization of imported amino acids and peptides as a carbon source. C) Quantification of CFUs with *H. halodurans* WT and Δ*ole-oapA* strains after 16 h incubation in MM using CasAAs [1% w/v] as the carbon source. Utilization of casamino acids does not require digestion by secreted proteases prior to import. D) Quantifications of CFUs with WT and Δ*ole*-*oapA* strains after 40 h incubation in MM using casein as the carbon source in the absence (−) or presence (+) of 10 µM Mn^2+^. Experiments in (B–D) were performed in biological triplicate where each replicate comprised of one technical replicate. Bars represent the average CFU mL^−1^ and error bars depict standard deviation.

The results of this initial milk agar experiment were consistent with the hypothesis that the Δ*ole-oapA* strain has a protein secretion deficiency compared to WT (Fig. 5A). Intriguingly, all carbon-source suppressor isolates examined appear to improve protein secretion of the Δ*ole-oapA* parent strain. Some suppressor isolates, such as *mntB* F146S, *ykoY* L82S, and *bmrC* V238*, even appear to supersede the ability of the WT strain to increase the transparency of milk agar. Given the proposed function of YkoY as a major contributor to the process of protein secretion (32), suppressor isolates carrying mutations in the *ykoY* riboswitch, the *ykoY* ORF, and *mntB* loci were expected to enhance protein secretion. This expectation is because YkoY expression should be increased by the suppressor mutations in the riboswitch or by increased concentrations of Mn^2+^ (Fig. 4). The mechanisms by which mutations to *phoR* or to components of *bmrCD* also improve protein secretion are less clear. However, PhoR is known to regulate the expression of secreted phosphatases (55) and might be linked to the expression of an exoprotease via PhoP under phosphate limitation conditions (56).

To verify that the milk agar assay results are exclusively due to differences in protein secretion levels and not growth rates, we sought additional data to confirm that protein secretion is relevant to OLE RNP complex function. Specifically, we evaluated the growth of *H. halodurans* in MM using casein protein as the carbon/energy source. In this assay, growth is limited by the need for bacteria to secrete proteases prior to the uptake of the resulting casein proteolysis products as amino acids and oligopeptides for use as carbon sources. Growth was measured by establishing the number of colony-forming units (CFUs) instead of optical density due to the opacity of media containing casein. Whereas the Δ*ole-oapA* strain struggles to grow on casein, all carbon-source suppressor isolates exhibit improved growth—with some strains exceeding that of WT cells (Fig. 5B). In contrast, WT and Δ*ole-oapA* cells exhibit near equivalent levels of growth when cultured with casamino acids (CasAAs), which are the product of acid-hydrolyzed casein (Fig. 5C). Thus, Δ*ole-oapA* cells grow normally if they do not need to secrete proteases to degrade casein.

The growth impairment of Δ*ole-oapA* cells when casein is the carbon/energy source, presumably caused by a protein secretion deficiency can also be overcome simply by the addition of Mn^2+^ to the medium (Fig. 5D). This result is consistent with the hypothesis that Mn^2+^ can boost the production of YkoY via the *ykoY* RS and that this protein participates in secretion of active proteases that degrade casein as part of the process to uptake amino acids as a food source. At this time, we cannot be certain that the OLE RNP complex influences protein secretion more generally, or if it is only involved in enabling the production and transport of active versions of the proteins required for casein utilization. Thus, additional studies will be needed to validate this proposed mechanism for enhancing the growth of bacteria lacking functional OLE RNP complexes in casein.

## Conclusion

The OLE RNP complex localizes to cell membranes (15), suggesting that part of its function involves biochemical processes relevant to the lipid bilayer and/or the cell wall. Genetic disruption of the OLE RNP complex was previously known to cause impaired growth under three apparently disparate stress conditions: cold, alcohol, and slightly elevated Mg^2+^ concentrations (22, 23). In the present study, we identified the fourth and fifth phenotypes associated with OLE RNA: poor growth with nonglucose carbon sources (Figs. 1 and 2) and diminished protein secretion (Fig. 5). These findings increase the remarkable diversity of fundamental processes proven to involve the OLE RNP complex.

It is possible that the carbon source and protein secretion phenotypes described here are related. When bacteria encounter unfavorable carbon/energy sources, the activities of major transcriptional factors such as CcpA and CodY are altered to promote the secretion of enzymes that break down external macromolecules for utilization as carbon sources (57–59). If disruption of the OLE RNP complex does result in a Mn^2+^ deficit when cells are grown in MM with gluconeogenic carbon sources, then the lack of YkoY protein might result in a greater proportion of “jammed” Sec translocons, which has been suggested to be the cause of the restricted growth in strains of *B. subtilis* lacking *ykoY* and its homolog *yceF* (32). If this mechanism is applicable to *H. halodurans*, however, it is unclear why excess Mg^2+^ in LB media would restrict the growth of cells when there is an abundance of other carbon/energy sources that do not require digestion. Further investigations into this proposed mechanism are necessary to assess its validity.

It remains to be determined if the OLE RNP complex functions to regulate protein secretion indirectly by managing the cell's Mn^2+^ levels or directly by interacting with the translocation protein machinery. Because OLE RNP complex function is also linked to the prevention of Mg^2+^ intoxication, and because OapA shares homology with Mg^2+^ transporters (23, 24), at present, it appears that the OLE RNP complex functions at least in part by regulating metal ion homeostasis. Indeed, metal ion homeostasis is proving to be a major feature of some growth inhibition phenotypes caused by OLE RNP complex disruption and of suppressor mutations that overcome these deleterious effects. The manipulation of cellular Mn^2+^ levels can overcome the Δ*ole-oapA* growth inhibition phenotypes caused by elevated Mg^2+^ and nonpreferred carbon sources (Fig. 3). However, growth inhibition by cold or ethanol stress fails to be relieved by manipulation of Mn^2+^ levels. These results suggest that several major cellular functions feature Mn^2+^ as a key metal cofactor or signaling agent. Therefore, Mn^2+^ homeostasis is unlikely to be the cause of all phenotypes observed when cells lack functional OLE RNP complexes. We also have evidence that an OapA mutant (16) that has been previously demonstrated to exhibit a more deleterious growth impairment than deletion of *ole* under the same stressful growth conditions fails to grow on any carbon source in the minimal medium utilized here, and this growth defect cannot be rescued by supplementation of Mn^2+^ to the media. These findings point to much broader biochemical roles for OLE RNA and the membrane-associated RNP particle it forms.

Indeed, we predict that many additional biological and biochemical processes will be relevant to the function of the OLE RNP complex. The organization of the *ole* gene cluster (14, 16, 24) hints that processes involving isoprenoid biosynthesis, DNA repair, ribosome assembly, and sporulation are also functionally or even physically linked to OLE RNA. Furthermore, OLE RNA pull-down experiments have yielded numerous additional protein candidates for the OLE RNP complex (19), raising the possibility that other physical associations between OLE RNA and proteins involved in fundamental biological processes occur. This diversity of known and prospective characteristics of the OLE RNP complex is consistent with the hypothesis (24) that this “bTOR” particle is the functional equivalent of mTOR complexes of eukaryotes.

## Materials and methods

### Bacterial growth conditions

All strains and plasmids used in this study are listed in Table S3. *Halalkalibacterium halodurans* strains were transformed with either pHCMC05 (60) or pHT254 (61) derivatives via protoplast transformation as described previously (13). These plasmids contain standard genetic elements such as chloramphenicol and ampicillin resistance genes, *lacI*, and *repA*. All *H. halodurans* C-125 (NC_002570) strains were grown in Lysogeny Broth Ultrapure (Thermo Fisher Scientific, Fair Lawn, NJ, USA) containing 1% [w/v] sodium carbonate hexahydrate such that the medium was pH 10. Unless stated otherwise, all growth media were supplemented with 3 μg mL^−1^ chloramphenicol (Cm) and 1 mM isopropyl-β-D-thiogalactopyranoside, and all cultures were grown at 37 °C with shaking at 220 rpm. MM were modified from TSS medium (40) to permit growth of *H. halodurans*. MM contained 100 mM sodium bicarbonate (pH 8.5 at ∼20 °C), 2.5 mM K_2_HPO_4_, 0.2% [w/v] NH_4_Cl, 0.02% [w/v] MgSO_4_-heptahydrate, 40 µg mL^−1^ FeCl_3_-hexahydrate, 40 µg mL^−1^ sodium citrate-dihydrate, and 20 mM of the indicated carbon source. Prior to any growth assay experiments, glycerol stocks of the *H. halodurans* strains were freshly streaked on LB (pH 10) agar supplemented with 3 μg mL^−1^ Cm at 37 °C. All *H. halodurans* strains grow slowly in MM regardless of the carbon source used, unless the cells also carried a plasmid that provided Cm resistance and 3 µg mL^−1^ Cm was supplemented to the media. The underlying reason for the benefit of Cm on cell growth is unclear. However, the growth benefit of Cm occurs with WT and mutant strains of *H. halodurans*. Furthermore, the carbon source phenotype and suppressor mutation effects are also observed in OLE RNP complex knockout strains without plasmids and without added Cm (see Tables S1 and S2). These findings indicate that the growth benefit derived by the addition of low doses of Cm is independent of the OLE RNP complex. Thus, experiments were conducted with strains of *H. halodurans* harboring empty plasmids and in the presence of 3 μg mL^−1^ Cm unless otherwise indicated.

### Growth assays in liquid culture

Bacteria were first grown overnight in 3 mL of LB (pH 10). The next morning, OD_600_ values of the overnight cultures were measured using a Cary 60 UV-Vis Spectrophotometer (Agilent Technologies, Santa Clara, CA, USA), and the cultures were then diluted to an OD_600_ of ∼0.05 in 3 mL of fresh LB (pH 10) medium and grown for 3 to 4 h. For growth assays in MM, the bacteria were then washed three times in phosphate-buffered saline (PBS; Gibco) to minimize LB contamination. Next, the OD_600_ of the cell cultures was measured (usually 0.5–0.8) and the cells were then diluted to an OD_600_ of 0.01 in the designated stress media: MM containing the indicated carbon source, LB (pH 10) (incubated at 18 °C to induce cold stress), LB (pH 10) with 10 mM MgCl_2_, or LB (pH 10) with 5.5% [v/v] ethanol. Unless stated otherwise, all growth assays were performed in biological triplicates wherein each biological replicate was comprised of three technical replicates.

MM experiments (Fig. 1C) were performed in 96-well round bottom plates with 200 µL culture per well. The plates were shaken (280 rpm) at 37 °C, and after 48 h, OD_600_ was measured using a BioTek Synergy Neo2 Multimode reader. Growth curve experiments (Figs. 1D and S7) were performed using a Bioscreen C instrument (Growth Curves USA, Piscataway, NJ, USA) with honeycomb plates (Bioscreen) with each well containing 200 µL of culture. Plates were incubated at 37 °C with maximum shaking and OD_600_ measurements were taken every 20 min. All other MM growth experiments were performed with 3 mL of media in 14 mL culture tubes with shaking (280 rpm) at 37 °C for 48 h unless indicated otherwise. At the designated time point, 200 µL of these cultures was aliquoted into wells of a flat-bottom 96-well plate (Costar #3788), and the OD_600_ was measured using a BioTek Synergy Neo2 Multimode reader.

Mg^2+^ and cold stress growth assays were performed with 3 mL of media in 14 mL culture tubes. After 1 and 4 days, respectively, 200 µL from each culture was measured using BioTek Synergy Neo2 Multimode reader as described above. Ethanol stress growth assays were performed in 96-well round bottom plates (Costar #3788) containing 100 μL culture per well. Importantly, plates were sealed with plate adhesive (Microseal “B” seals MSB1001, BioRad, Kidlington, UK) to prevent evaporation of ethanol. After 2 days, the OD_600_ was determined using the plate reader as described above.

### Agar spot assays

A total of 2.5 μL of overnight cultures were directly spotted onto agar supplemented with 3 μg mL^−1^ Cm. When the media component of the agar was either LB (pH 10) (for cold stress) or LB (pH 10) supplemented with 10 mM MgCl_2_, the agar concentration was 1.5% [w/v]. We noticed that contaminants in certain regular agars allowed growth of the Δ*ole-oapA* strain on EMM. Thus, for EMM agar and milk agar [5% [w/v] nonfat dry milk (LabScientific), 20 mM CHES (pH 10), and 200 mM NaCl], Nobel agar, ultrapure (Thermo Fisher Scientific) at a final concentration of 0.6 and 1% [w/v], respectively, was used. Images were acquired using the ProtoCOL3 plate imager (Synbiosis USA, Frederick, MD, USA).

### Genetic selections for suppressor mutations

Liquid cultures for genetic selections were performed with 3 mL of EMM as detailed above except that cultures were incubated until they became turbid (typically after 11–14 days). Once cultures appeared turbid, a glycerol stock was prepared and then streaked onto agar. A single colony was then used to inoculate a liquid culture for subsequent cryo-conservation and genomic DNA isolation. WGS was performed, and mutations were identified as previously described (23).

We observed that the parent Δ*ole* strain had acquired a background mutation that impacted growth even under standard conditions in LB (pH 10), and, consequently, all Δ*ole* EMM suppressor isolates also contained this background mutation (Table S2). Despite this background mutation, substantial overlap was observed in the mutated genes of the suppressor strains identified (Fig. S3).

### CFU assays

CFU assays were performed to determine the growth of *H. halodurans* in MM with casein and casamino acids as carbon sources due to the interference of casein with photometric measurements. The composition of the MM used was the same as described above except that the concentrations of casein and casamino acids were both 1% [w/v]. Growth assays were performed with 3 mL of media and incubated at 37 °C with shaking (220 rpm). After 40 h incubation, serial dilutions (10^0^ to 10^−5^) were prepared in PBS and 10 μL of each dilution was spotted onto LB (pH 10) agar containing 3 µg mL^−1^ Cm. Plates were subsequently incubated overnight at 37 °C. Colonies were manually counted from the two highest dilutions displaying colonies to derive the CFU mL^−1^ of the undiluted cultures. The reported CFU mL^−1^ represents the average CFU mL^−1^ from the two highest dilutions. In these assays, biological replicates are comprised of a single technical replicate.

### In vitro transcription termination assays

The transcription termination assays were conducted by adapting a previously established method for single-round transcription (62). DNA templates, including the native promoter from *H. halodurans* and the downstream region encompassing the *ykoY* riboswitch (Fig. 4A), were generated by polymerase chain reaction using Phusion High-Fidelity DNA Polymerase [New England Biolabs (NEB), Ipswich, MA, USA]. The resulting double-stranded DNA templates were purified using the QIAquick PCR Purification Kit (Qiagen, Venlo, The Netherlands) according to the manufacturer's instructions. DNA template and primer sequences are detailed in Table S4.

Transcription terminations were prepared by first incubating 100 nM DNA template in transcription buffer (20 mM Tris-HCl [pH 8.0 at ∼20 °C], 20 mM NaCl, 100 µM EDTA, 0.01 mg mL^−1^ BSA, 1% [v/v] glycerol) plus 135 µM of the dinucleotide ApA, 0.04 U µL^−1^*E. coli* RNA polymerase (NEB), cytosine triphosphate (CTP), adenosine triphosphate (ATP) (2.5 µM each), uridine triphosphate (UTP) (1.0 µM), and [α-^32^P]-UTP (2 µCi). At this step, divalent ions were also added to the reaction mixture such that the final concentration in the assay was either 1.2 mM MgCl_2_ (Fig. 4B and C, −lane) or 1 mM MgCl_2_ and 0.2 mM MnCl_2_ (Fig. 4B and C, +lane). This initial reaction mixture, which comprised nine-tenths of the final reaction volume, was incubated for 20 to 30 min at 37 °C to allow the RNA polymerase to stall at the first G residue of the transcript, which is six nucleotides downstream from the transcription start site. The stalled complexes resumed transcription with the addition of one-tenth reaction volume of an elongation buffer composed of the aforementioned transcription buffer supplemented with 1.5 mM each of ATP, CTP, guanosine triphosphate, 0.5 mM UTP, and 1 mg mL^−1^ heparin. The mixture was incubated for 30 min at 37 °C and then quenched by the addition of an equal volume of a loading solution (8M urea, 20% [w/v] sucrose, 0.1% [w/v] SDS, 0.05% [w/v] bromophenol blue, 0.05% [w/v] xylene cyanol, 0.09 M Tris, 0.09 M borate, and 100 mM EDTA).

Reaction products were separated by denaturing 10% polyacrylamide gel electrophoresis (PAGE) and visualized using a Typhoon FLA 9500 Molecular Scanner (GE Healthcare, Chicago, IL, USA). Band intensities were quantified using ImageQuant TL software v8.1 (Cytiva, Hauppauge, NY, USA). Transcription termination assays were performed in triplicate, and a representative of the resulting PAGE autoradiogram is presented (Fig. 4B and C). Using band intensities, the amounts of FL and terminated (T) transcripts were estimated by accounting for the different number of U residues in each transcript, and the average with standard deviation of the percentage of FL transcript of all three replicates is specified for each condition below the PAGE autoradiograms.

## Supplementary Material

pgae075_Supplementary_Data

## Data Availability

All experimental data are included in the main text and SI Appendix.

## References

[1] Nissen P , Hansen J, Ban N, Moore PB, Steitz TA. 2000. The structural basis of ribosome activity in peptide bond synthesis. Science. 289:920–930. 10937990 10.1126/science.289.5481.920

[2] Guerrier-Takada C , Gardiner K, Marsh T, Pace N, Altman S. 1983. The RNA moiety of ribonuclease P is the catalytic subunit of the enzyme. Cell. 35:849–857. 6197186 10.1016/0092-8674(83)90117-4

[3] Janssen BD , Hayes CS. 2012. The tmRNA ribosome-rescue system. Adv Protein Chem Struct Biol. 86:151–191. 22243584 10.1016/B978-0-12-386497-0.00005-0 PMC3358797

[4] Keiler KC , Waller PRH, Sauer RT. 1996. Role of a peptide tagging system in degradation of proteins synthesized from damaged messenger RNA. Science. 271:990–993. 8584937 10.1126/science.271.5251.990

[5] Poritz MA , Strub K, Walter P. 1988. Human SRP RNA and *E. coli* 4.5S RNA contain a highly homologous structural domain. Cell. 55:4–6. 2458843 10.1016/0092-8674(88)90003-7

[6] Cech TR . 1990. Self-splicing of group I introns. Annu Rev Biochem. 59:543–568. 2197983 10.1146/annurev.bi.59.070190.002551

[7] Zimmerly S , Hausner G, Wu X. 2001. Phylogenetic relationships among group II intron ORFs. Nucleic Acids Res. 29:1238–1250. 11222775 10.1093/nar/29.5.1238 PMC29734

[8] Weinberg Z , et al 2017. Detection of 224 candidate structured RNAs by comparative analysis of specific subsets of intergenic regions. Nucleic Acids Res. 45:10811–10823. 28977401 10.1093/nar/gkx699 PMC5737381

[9] Harris KA , Breaker RR. 2018. Large noncoding RNAs in bacteria. Microbiol Spectr. 6:10.1128/microbiolspec.RWR-0005-2017. 10.1128/microbiolspec.rwr-0005-2017 PMC6042979 29992899

[10] Gilbert W . 1986. The RNA world. Nature. 319:618.

[11] Joyce GF . 1989. RNA evolution and the origins of life. Nature. 338:217–224. 2466202 10.1038/338217a0

[12] Joshi A , Thite S, Karodi P, Joseph N, Lodha T. 2021. *Alkalihalobacterium elongatum* gen. nov. sp. nov.: an antibiotic-producing bacterium isolated from Lonar Lake and reclassification of the genus *Alkalihalobacillus* into seven novel genera. Front Microbiol. 12:722369. 34707580 10.3389/fmicb.2021.722369 PMC8543038

[13] Wallace JG , Breaker RR. 2011. Improved genetic transformation methods for the model alkaliphile *Bacillus halodurans* C-125. Lett Appl Microbiol. 52:430–432. 21362000 10.1111/j.1472-765X.2011.03017.x PMC5315388

[14] Puerta-Fernandez E , Barrick JE, Roth A, Breaker RR. 2006. Identification of a large noncoding RNA in extremophilic eubacteria. Proc Natl Acad Sci USA. 103:19490–19495. 17164334 10.1073/pnas.0607493103 PMC1748253

[15] Block KF , Puerta-Fernandez E, Wallace JG, Breaker RR. 2011. Association of OLE RNA with bacterial membranes via an RNA-protein interaction. Mol Microbiol. 79:21–34. 21166891 10.1111/j.1365-2958.2010.07439.x PMC3552494

[16] Harris KA , Zhou Z, Peters ML, Wilkins SG, Breaker RR. 2018. A second RNA-binding protein is essential for ethanol tolerance provided by the bacterial OLE ribonucleoprotein complex. Proc Natl Acad Sci U S A. 115:E6319–E6328. 29915070 10.1073/pnas.1803191115 PMC6142196

[17] Widner DL , Harris KA, Corey L, Breaker RR. 2020. *Bacillus halodurans* OapB forms a high-affinity complex with the P13 region of the noncoding RNA OLE. J Biol Chem. 295:9326–9334. 32376692 10.1074/jbc.RA120.012676 PMC7363126

[18] Yang Y , Harris KA, Widner DL, Breaker RR. 2021. Structure of a bacterial OapB protein with its OLE RNA target gives insights into the architecture of the OLE ribonucleoprotein complex. Proc Natl Acad Sci U S A. 118:e2020393118. 10.1073/pnas.2020393118 PMC7936274 33619097

[19] Lyon SE , Harris KA, Odzer NB, Wilkins SG, Breaker RR. 2022. Ornate, large, extremophilic (OLE) RNA forms a kink turn necessary for OapC protein recognition and RNA function. J Biol Chem. 298:102674. 36336078 10.1016/j.jbc.2022.102674 PMC9723947

[20] Baird NJ , Zhang J, Hamma T, Ferré-D’Amaré AR. 2012. Ybxf and YlxQ are bacterial homologs of L7Ae and bind K-turns but not K-loops. RNA. 18:759–770. 22355167 10.1261/rna.031518.111 PMC3312563

[21] Kyrpides NC , Woese CR, Ouzounis CA. 1996. KOW: a novel motif linking a bacterial transcription factor with ribosomal proteins. Trends Biochem Sci. 21:425–426. 8987397 10.1016/s0968-0004(96)30036-4

[22] Wallace JG , Zhou Z, Breaker RR. 2012. OLE RNA protects extremophilic bacteria from alcohol toxicity. Nucleic Acids Res. 40:6898–6907. 22561371 10.1093/nar/gks352 PMC3413148

[23] Harris KA , Odzer NB, Breaker RR. 2019. Disruption of the OLE ribonucleoprotein complex causes magnesium toxicity in *Bacillus halodurans*. Mol Microbiol. 112:1552–1563. 31461569 10.1111/mmi.14379 PMC6842403

[24] Breaker RR , Harris KA, Lyon SE, Wencker FDR, Fernando CM. 2023. Evidence that OLE RNA is a component of a major stress-responsive ribonucleoprotein particle in extremophilic bacteria. Mol Microbiol. 120:324–340. 37469248 10.1111/mmi.15129

[25] Saxton RA , Sabatini DM. 2017. mTOR signaling in growth, metabolism, and disease. Cell. 168:960–976. 28283069 10.1016/j.cell.2017.02.004 PMC5394987

[26] Liu GY , Sabatini DM. 2020. mTOR at the nexus of nutrition, growth, ageing and disease. Nat Rev Mol Cell Biol. 21:183–203. 31937935 10.1038/s41580-019-0199-y PMC7102936

[27] Czaplewski LG , North AK, Smith MCM, Baumberg S, Stockley PG. 1992. Purification and initial characterization of AhrC: the regulator of arginine metabolism genes in *Bacillus subtilis*. Mol Microbiol. 6:267–275. 1312212 10.1111/j.1365-2958.1992.tb02008.x

[28] Klingel U , Miller CM, North AK, Stockley PG, Baumberg S. 1995. A binding site for activation by the *Bacillus subtilis* AhrC protein, a repressor/activator of arginine metabolism. Mol Gen Genet. 248:329–340. 7565595 10.1007/BF02191600

[29] Barrick JE , et al 2004. New RNA motifs suggest an expanded scope for riboswitches in bacterial genetic control. Proc Natl Acad Sci U S A. 101:6421–6426. 15096624 10.1073/pnas.0308014101 PMC404060

[30] Dambach M , et al 2015. The ubiquitous *yybP-ykoY* riboswitch is a manganese-responsive regulatory element. Mol Cell. 57:1099–1109. 25794618 10.1016/j.molcel.2015.01.035 PMC4376352

[31] Price IR , Gaballa A, Ding F, Helmann JD, Ke A. 2015. Mn^2+^-sensing mechanisms of *yybP-ykoY* orphan riboswitches. Mol Cell. 57:1110–1123. 25794619 10.1016/j.molcel.2015.02.016 PMC4703321

[32] He B , Sachla AJ, Helmann JD. 2023. TerC proteins function during protein secretion to metalate exoenzymes. Nat Commun. 14:6186. 37794032 10.1038/s41467-023-41896-1 PMC10550928

[33] Paruthiyil S , Pinochet-Barros A, Huang X, Helmann JD. 2020. *Bacillus subtilis* TerC family proteins help prevent manganese intoxication. J Bacteriol. 202:e00624-19. 31685536 10.1128/JB.00624-19 PMC6941523

[34] Rocha EPC . 2008. The organization of the bacterial genome. Annu Rev Genet. 42:211–233. 18605898 10.1146/annurev.genet.42.110807.091653

[35] Ogura M , Matsutani M, Asai K, Suzuki M. 2023. Glucose controls manganese homeostasis through transcription factors regulating known and newly identified manganese transporter genes in *Bacillus subtilis*. J Biol Chem. 299:105069. 37468100 10.1016/j.jbc.2023.105069 PMC10448178

[36] Heidrich N , Moll I, Brantl S. 2007. *In vitro* analysis of the interaction between the small RNA SR1 and its primary target *ahrC* mRNA. Nucleic Acids Res. 35:4331–4346. 17576690 10.1093/nar/gkm439 PMC1935000

[37] Licht A , Preis S, Brantl S. 2005. Implication of CcpN in the regulation of a novel untranslated RNA (SR1) in *Bacillus subtilis*. Mol Microbiol. 58:189–206. 16164558 10.1111/j.1365-2958.2005.04810.x

[38] Gimpel M , Brantl S. 2016. Dual-function sRNA encoded peptide SR1P modulates moonlighting activity of *B. subtilis* GapA. RNA Biol. 13:916–926. 27449348 10.1080/15476286.2016.1208894 PMC5013986

[39] Julsing MK , Rijpkema M, Woerdenbag HJ, Quax WJ, Kayser O. 2007. Functional analysis of genes involved in the biosynthesis of isoprene in *Bacillus subtilis*. Appl Microbiol Biotechnol. 75:1377–1384. 17458547 10.1007/s00253-007-0953-5 PMC1914294

[40] Fouet A , Sonenshein AL. 1990. A target for carbon source-dependent negative regulation of the *citB* promoter of *Bacillus subtilis*. J Bacteriol. 172:835–844. 2105305 10.1128/jb.172.2.835-844.1990 PMC208513

[41] Walker MC , van der Donk WA. 2016. The many roles of glutamate in metabolism. J Ind Microbiol Biotechnol. 43:419–430. 26323613 10.1007/s10295-015-1665-y PMC4753154

[42] Wilson KS , von Hippel PH. 1995. Transcription termination at intrinsic terminators: the role of the RNA hairpin. Proc Natl Acad Sci U S A. 92:8793–8797. 7568019 10.1073/pnas.92.19.8793 PMC41053

[43] Yarnell WS , Roberts JW. 1999. Mechanism of intrinsic transcription termination and antitermination. Science. 284:611–615. 10213678 10.1126/science.284.5414.611

[44] McCown PJ , Corbino KA, Stav S, Sherlock ME, Breaker RR. 2017. Riboswitch diversity and distribution. RNA. 23:995–1011. 28396576 10.1261/rna.061234.117 PMC5473149

[45] Kavita K , Breaker RR. 2023. Discovering riboswitches: the past and the future. Trends Biochem Sci. 48:119–141. 36150954 10.1016/j.tibs.2022.08.009 PMC10043782

[46] Que Q , Helmann JD. 2000. Manganese homeostasis in *Bacillus subtilis* is regulated by MntR, a bifunctional regulator related to the diphtheria toxin repressor family of proteins. Mol Microbiol. 35:1454–1468. 10760146 10.1046/j.1365-2958.2000.01811.x

[47] Shi L , Hulett FM. 1999. The cytoplasmic kinase domain of PhoR is sufficient for the low phosphate-inducible expression of Pho regulon genes in *Bacillus subtilis*. Mol Microbiol. 31:211–222. 9987123 10.1046/j.1365-2958.1999.01163.x

[48] Torres C , Galián C, Freiberg C, Fantino JR, Jault JM. 2009. The YheI/YheH heterodimer from *Bacillus subtilis* is a multidrug ABC transporter. Biochim Biophys Acta. 1788:615–622. 19167342 10.1016/j.bbamem.2008.12.012

[49] Thaker TM , et al 2022. Asymmetric drug binding in an ATP-loaded inward-facing state of an ABC transporter. Nat Chem Biol. 18:226–235. 34931066 10.1038/s41589-021-00936-x PMC9242650

[50] Reilman E , Mars RAT, van Dijl JM, Denham EL. 2014. The multidrug ABC transporter BmrC/BmrD of *Bacillus subtilis* is regulated via a ribosome-mediated transcriptional attenuation mechanism. Nucleic Acids Res. 42:11393–11407. 25217586 10.1093/nar/gku832 PMC4191407

[51] Fukushima S , Yoshimura M, Chibazakura T, Sato T, Yoshikawa H. 2006. The putative ABC transporter YheH/YheI is involved in the signalling pathway that activates KinA during sporulation initiation. FEMS Microbiol Lett. 256:90–97. 16487324 10.1111/j.1574-6968.2006.00104.x

[52] Kehres DG , Maguire ME. 2003. Emerging themes in manganese transport, biochemistry and pathogenesis in bacteria. FEMS Microbiol Rev. 27:263–290. 12829271 10.1016/S0168-6445(03)00052-4

[53] Oh YK , Freese E. 1976. Manganese requirement of phosphoglycerate phosphomutase and its consequences for growth and sporulation of *Bacillus subtilis*. J Bacteriol. 127:739–746. 182667 10.1128/jb.127.2.739-746.1976 PMC232979

[54] Morris LS , Evans J, Marchesi JR. 2012. A robust plate assay for detection of extracellular microbial protease activity in metagenomic screens and pure cultures. J Microbiol Methods. 91:144–146. 22921428 10.1016/j.mimet.2012.08.006

[55] Hulett FM , et al 1994. Sequential action of two-component genetic switches regulates the PHO regulon in *Bacillus subtilis*. J Bacteriol. 176:1348–1358. 8113174 10.1128/jb.176.5.1348-1358.1994 PMC205199

[56] Harwood CR , Kikuchi Y. 2022. The ins and outs of *Bacillus* proteases: activities, functions and commercial significance. FEMS Microbiol Rev. 46:fuab046. 34410368 10.1093/femsre/fuab046 PMC8767453

[57] Kim JH , Guvener ZT, Cho JY, Chung KC, Chambliss GH. 1995. Specificity of DNA binding activity of the *Bacillus subtilis* catabolite control protein CcpA. J Bacteriol. 177:5129–5134. 7665492 10.1128/jb.177.17.5129-5134.1995 PMC177293

[58] Barbieri G , et al 2015. CodY regulates expression of the *Bacillus subtilis* extracellular proteases Vpr and Mpr. J Bacteriol. 197:1423–1432. 25666135 10.1128/JB.02588-14 PMC4372738

[59] Bulock LL , et al 2022. Interplay of CodY and CcpA in regulating central metabolism and biofilm formation in *Staphylococcus aureus*. J Bacteriol. 204:e00617-21. 35735992 10.1128/jb.00617-21 PMC9295537

[60] Nguyen HD , et al 2005. Construction of plasmid-based expression vectors for *Bacillus subtilis* exhibiting full structural stability. Plasmid. 54:241–248. 16005967 10.1016/j.plasmid.2005.05.001

[61] Phan TTP , Tran LT, Schumann W, Nguyen HD. 2015. Development of Pgrac100-based expression vectors allowing high protein production levels in *Bacillus subtilis* and relatively low basal expression in *Escherichia coli*. Microb Cell Fact. 14:72. 25990516 10.1186/s12934-015-0255-z PMC4446860

[62] Landick R , Wang D, Chan CL. 1996. Quantitative analysis of transcriptional pausing by *Escherichia coli* RNA polymerase: *his* leader pause site as paradigm. Methods Enzymol. 274:334–353. 8902817 10.1016/s0076-6879(96)74029-6

