# Supplementary material for: Disruption of the bacterial OLE RNP complex impairs growth on alternative carbon sources
Source: PNAS Nexus. 2024 Feb 12;3(2):pgae075. doi: 10.1093/pnasnexus/pgae075 (PMC10898510; doi:10.1093/pnasnexus/pgae075)
Supplement: pgae075_Supplementary_Data [file pgae075_supplementary_data.zip › PNASNEXUS-PNASNEXUS-2023-01344R-s02.docx]

**Supplemental Information**

**Disruption of the bacterial OLE RNP complex impairs growth on alternative carbon sources**

Seth E. Lyon^a,1^, Freya D. R. Wencker^b,1^, Chrishan M. Fernando^a^, Kimberly A. Harris^c,d^, and Ronald R. Breaker^a,b,c,2^

^a^Department of Molecular Biophysics and Biochemistry, Yale University, New Haven, CT 06511-8103, USA;

^b^Howard Hughes Medical Institute, Yale University, New Haven, CT 06511-8103, USA;

^c^Department of Molecular, Cellular and Developmental Biology, Yale University, New Haven, CT 06511-8103, USA

^d^Present address: Tessera Therapeutics, Somerville, MA, USA.

^1^S.E.L. and F.D.R.W. contributed equally to this work.

^2^To whom correspondence may be addressed. e-mail: [ronald.breaker@yale.edu](mailto:ronald.breaker@yale.edu)

**
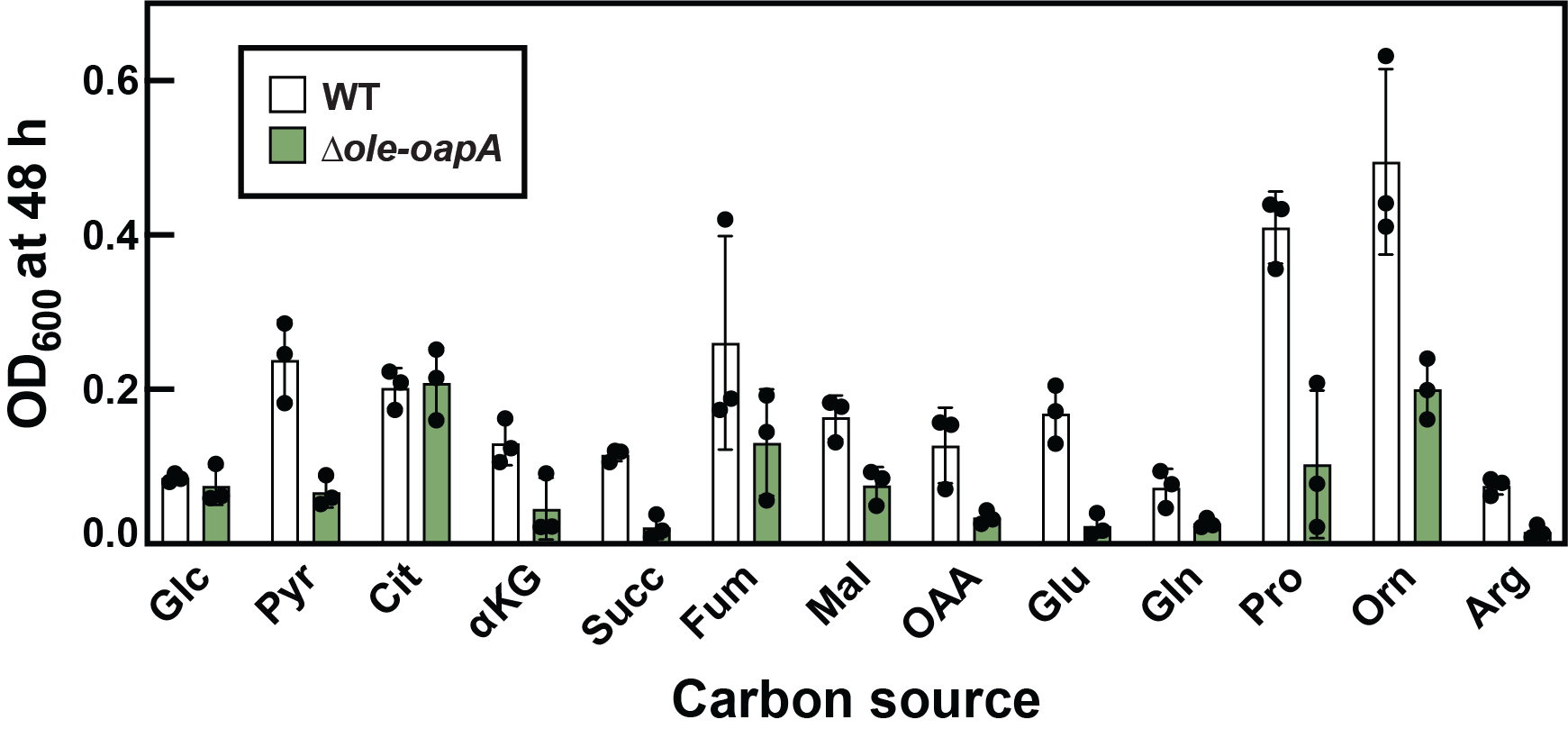
**

**Fig. S1.** OLE RNP complex function facilitates growth on alternative carbon sources. WT (open) and ∆*ole-oapA* (green) *H. halodurans* strains were grown in minimal media with the indicated carbon sources in 96-well round-bottom plates as described in the Methods section. This graph depicts the OD_600_ values that were used to normalize growth of *H. halodurans* ∆*ole-oapA* to WT in **Fig. 1*C***. Bars represent the average OD_600_ from three biological replicates wherein each replicate was composed of three technical replicates. Error bars represent standard deviation.

**
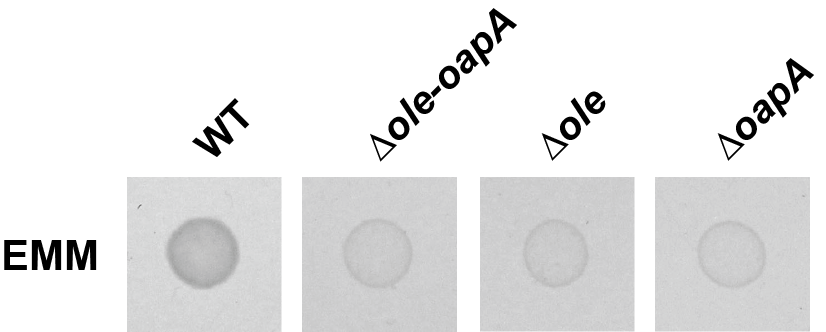
**

**Fig. S2.** Robust growth of *H. halodurans* in EMM depends on a functional OLE RNP complex. Shown is a representative image of an EMM agar spot assay with the WT, ∆*ole-oapA*, ∆*ole*, ∆*oapA* strains of *H. halodurans*. The image was recorded after 22 h of growth at 37°C.


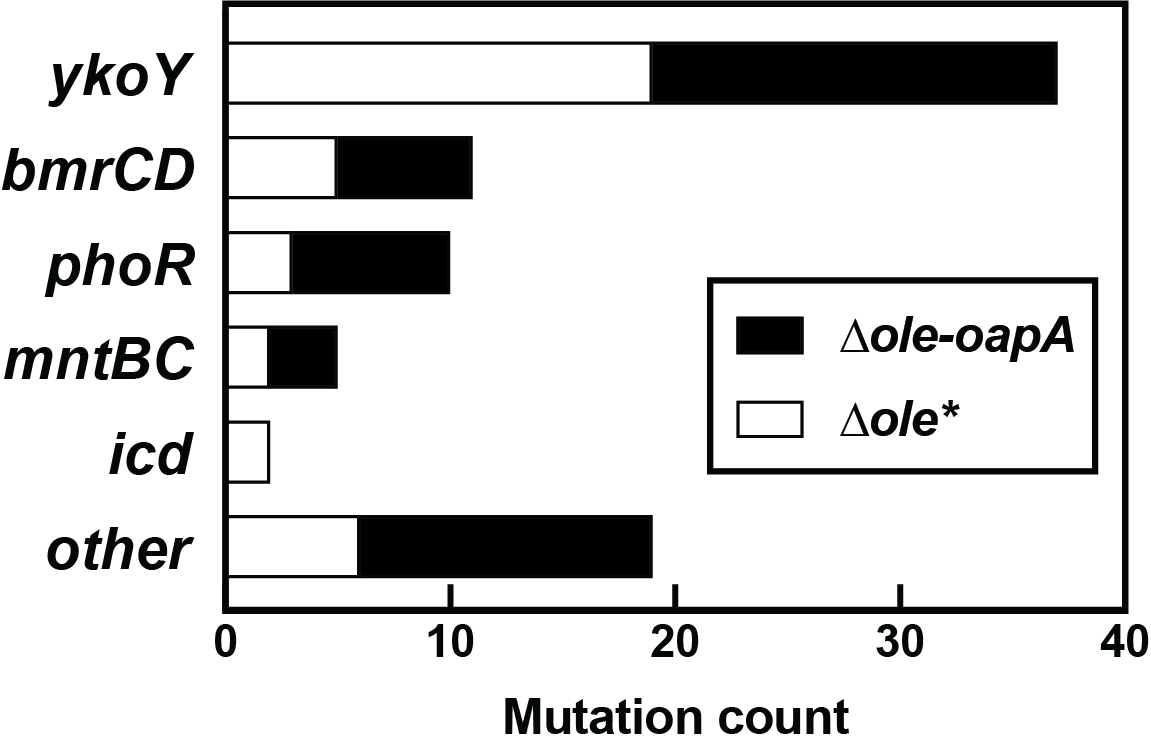


**Fig. S3.** Common suppressor mutations are identified in ∆*ole* and ∆*ole-oapA* strains of *H. halodurans*. A plot of the number of mutations observed in carbon-source genetic suppressor selections. The asterisk indicates all strains contained a background mutation in *ptsP*.


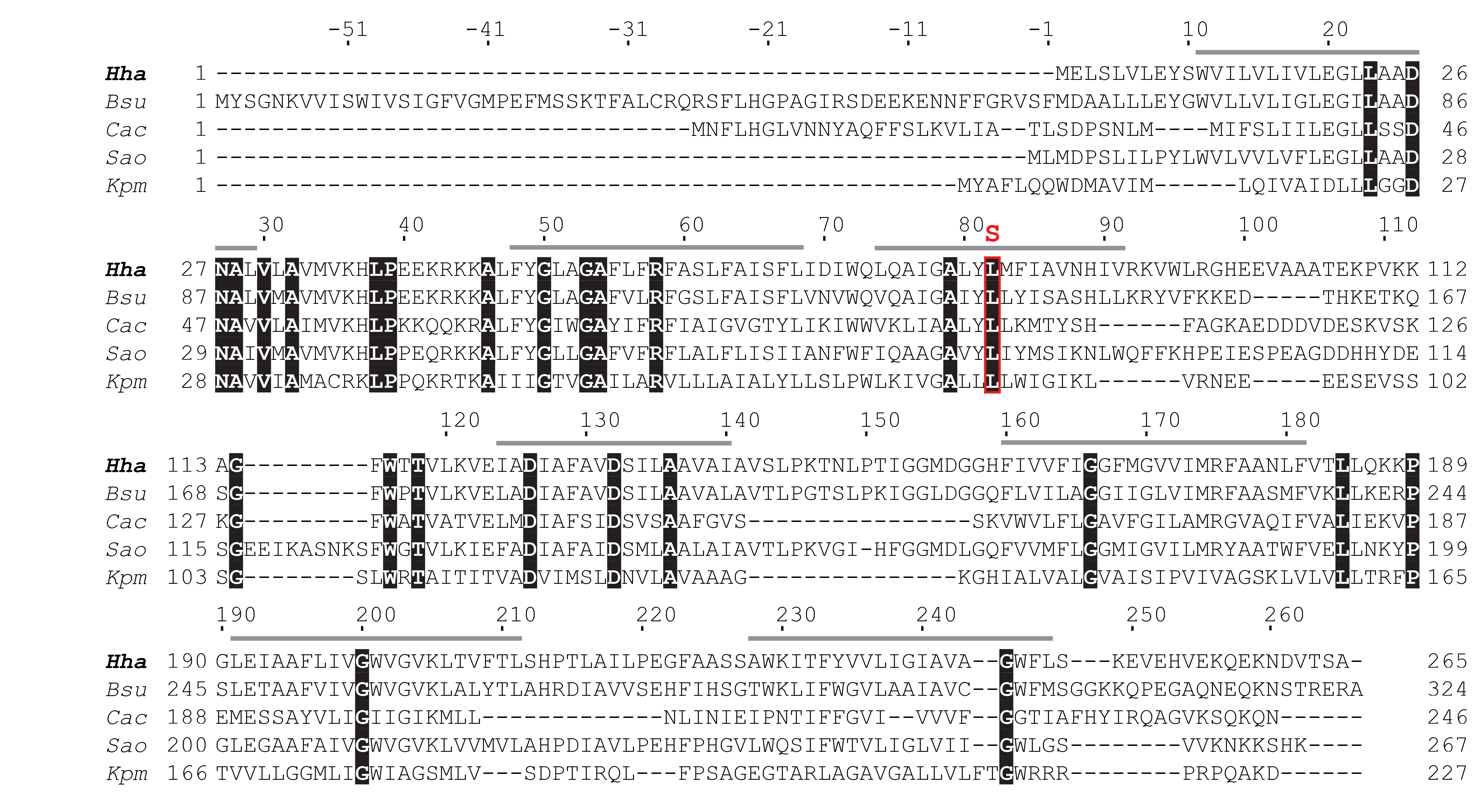


**Fig. S4.** The amino acid residue L82 in YkoY of *H. halodurans* is highly conserved. Multiple sequence alignment of *H. halodurans* YkoY (Hha, WP_010898704.1) with homologs from *B. subtilis* (Bsu, WP_009967096.1), *Clostridium acetobutylicum* (Cac, WP_010964724.1), *Staphylococcus aureus* (Sao, WP_000928413.1), and *Klebsiella pneumoniae* (Kpm, WP_004199298.1). Fully conserved amino acids are highlighted in black, and the numbers (bottom right) report the protein lengths. The highly conserved leucine residues corresponding to L82 in Hha YkoY are highlighted with a red box. The mutation L82S observed in the EMM genetic selections with *H. halodurans* is indicated with a red S above the amino acid sequences. Predicted transmembrane domains of the *H. halodurans* YkoY are indicated by gray bars above the amino acid sequences. Topology was predicted using DeepTMHMM (<https://dtu.biolib.com/DeepTMHMM>).

**
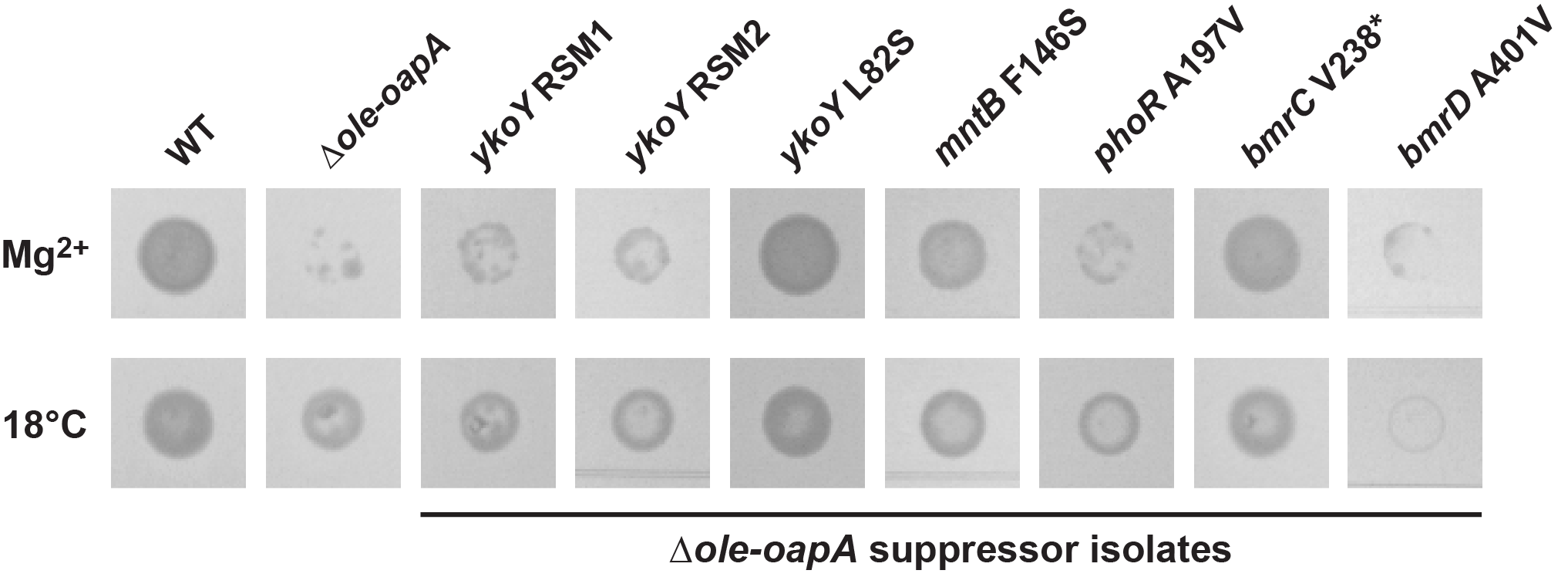
**

**Fig. S5.** Agar spot assays of ∆*ole-oapA* carbon-source suppressor isolates under Mg^2+^ and cold stresses. Depicted are representative images of agar spot assays with the WT, ∆*ole-oapA*, and ∆*ole-oapA* carbon-source suppressor isolates of *H. halodurans*. Top: Mg^2+^ (10 mM) stress after one day of growth. Bottom: Cold (18°C) stress after four days of growth.

**
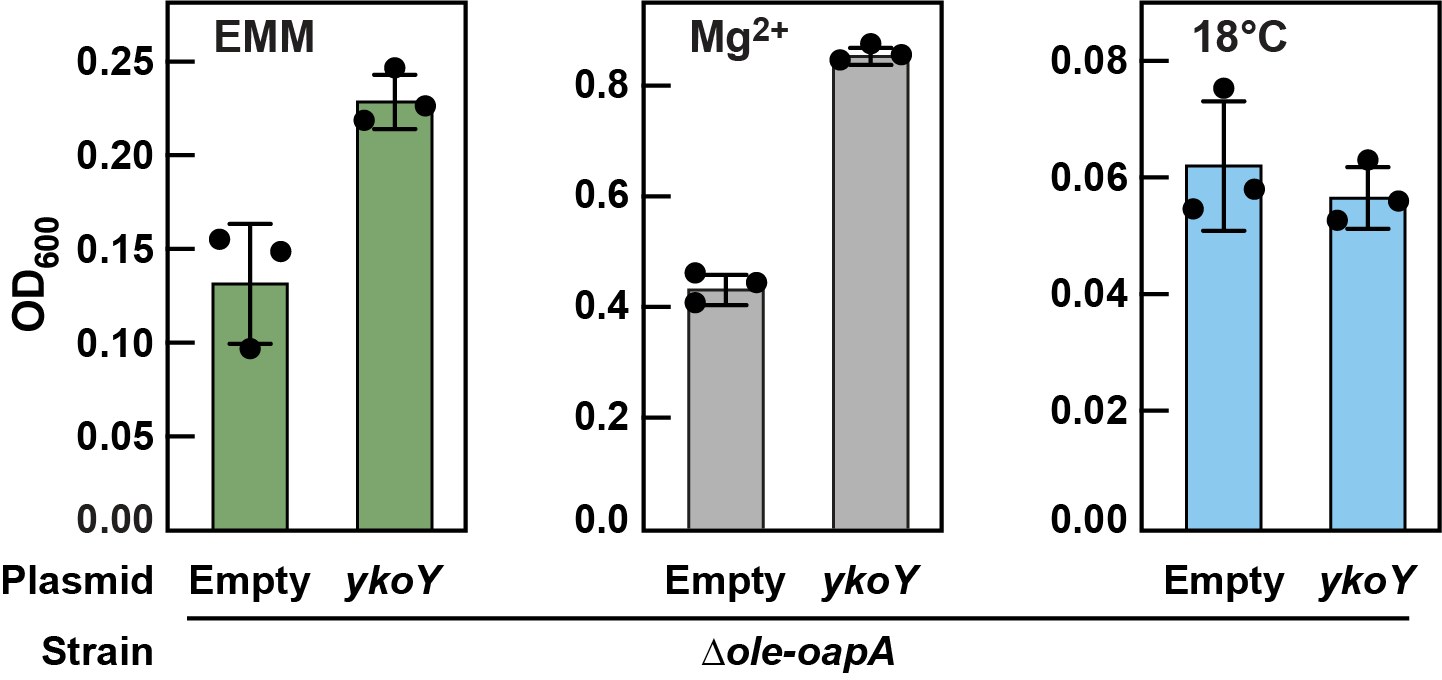
**

**Fig. S6.** Overexpression of *ykoY* in *H. halodurans* ∆*ole-oapA* cells improves growth under EMM and Mg^2+^ stresses. *H. halodurans* ∆*ole-oapA* cells were grown in the presence of 1 mM IPTG to induce expression of the *ykoY* gene prior to and during growth under the indicated stress condition. OD_600_ measurements were recorded at 48 h for EMM (left), 24 h for Mg^2+^ (10 mM) stress (middle), and 72 h for cold (18°C) stress (right). Bars represent the average OD_600_ from three biological replicates wherein each replicate was composed of three technical replicates. Error bars represent standard deviation.

**
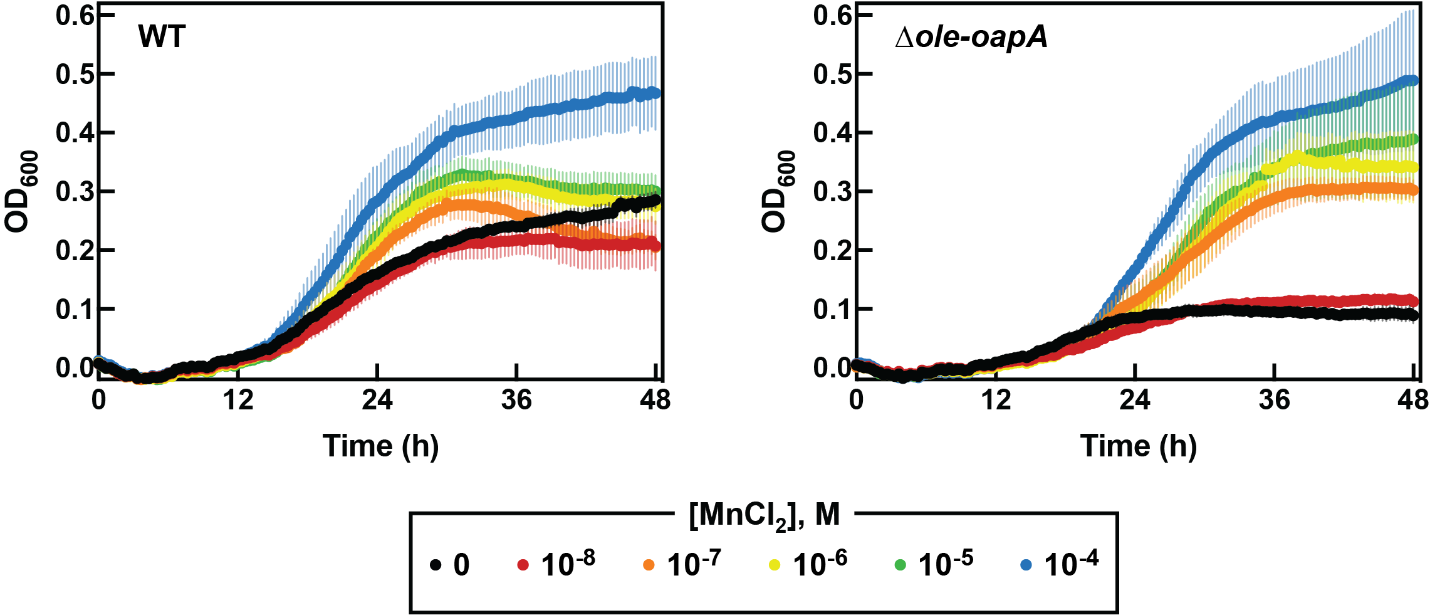
**

**Fig. S7.** Mn^2+^ supplementation rescues growth of *H. halodurans* ∆*ole-oapA* in EMM. Growth curves of *H. halodurans* WT (left) and ∆*ole-oapA* (right) in EMM with increasing concentrations of MnCl_2_. These experiments were performed identically to **Fig. 1*D*** as described in the Methods section. Each data point represents the average OD_600_ from three biological replicates wherein each replicate consisted of three technical replicates. Error bars represent standard deviation.

**
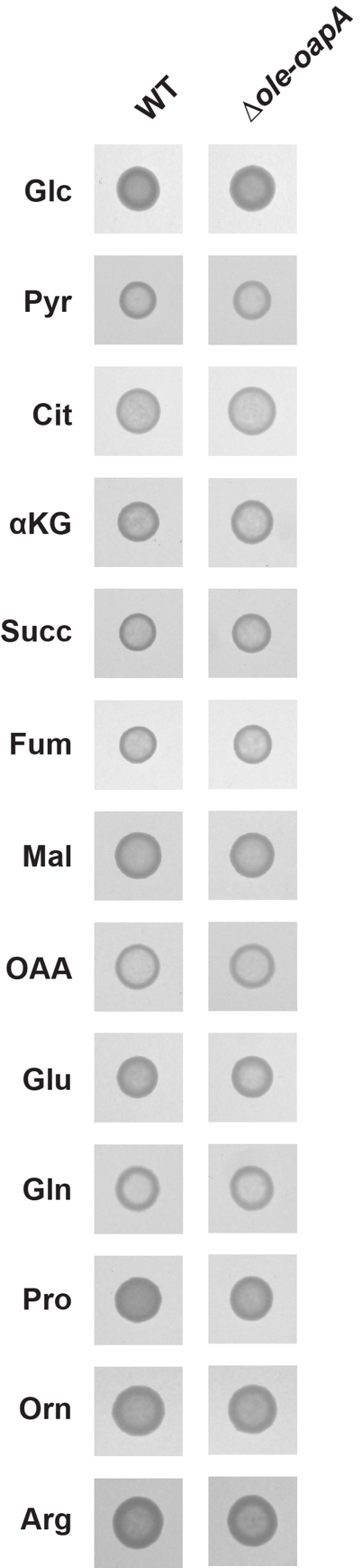
**

**Fig. S8.** Supplementation of Mn^2+^ rescues growth of *H. halodurans* ∆*ole-oapA* cells in minimal media with all carbon sources. Representative agar spot assay with WT and ∆*ole-oapA* *H. halodurans* cells that were grown on minimal media agar with the indicated carbon source supplemented with 10 µM MnCl_2_. The images were recorded after 48 h of growth at 37°C.

**
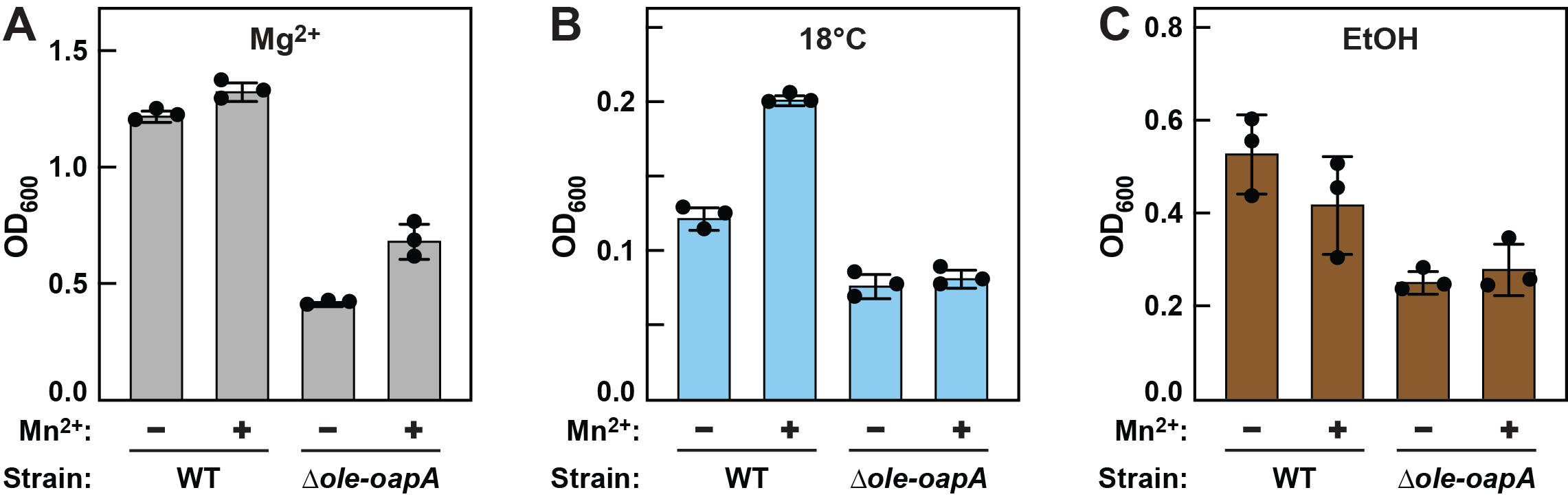
**

**Fig. S9.** Supplementation of Mn^2+^ improves growth of *H. halodurans* ∆*ole-oapA* under Mg^2+^ stress, but not under either cold or ethanol stresses. WT and ∆*ole-oapA* strains of *H. halodurans* were grown in LB (pH 10) under the indicated stress condition in the absence (–) or presence (+) of 10 µM MnCl_2_. (**A)** Mg^2+^ (10 mM) stress. (**B)** Cold (18°C) stress. (**C)** Ethanol [5% v/v] stress. All growth assays were performed using 3 mL cultures in 14 mL culture tubes. Bars represent the average OD_600_ from three biological replicates wherein each replicate was composed of three technical replicates. Error bars represent standard deviation.

**Table S3.** *H. halodurans* C-125 strains used in this study. Note: "Genotype" indicates the status of the bacterial chromosome.

| Name | Genotype | Plasmid Name | Reference |
| --- | --- | --- | --- |
| WT | Wild type | pHCMC05 empty | i |
| ∆*ole-oapA* | ∆*ole-oapA* | - | i |
| ∆*ole-oapA* | ∆*ole-oapA* | pHCMC05 empty | i |
| ∆*ole* | ∆*ole* | - | i |
| ∆*ole* | ∆*ole* | pHCMC05 empty | this study |
| ∆*oapA* | ∆*oapA* | pHCMC05 empty | this study |
| ∆*ole-oapA* | ∆*ole-oapA* | pHT254 empty | this study |
| ∆*ole-oapA* | ∆*ole-oapA* | pHT254 ykoY | this study |

**i)** J. G. Wallace, Z. Zhou, R. R. Breaker, OLE RNA protects extremophilic bacteria from alcohol toxicity. *Nucleic Acids Res.* **40**, 6898–6907 (2012).

**Table S4.** DNA templates and primers used in this study

| Name | Sequence | Purpose |
| --- | --- | --- |
| Bha_ykoY_ RS_DNA_ template | TAAAAAATGGTTGACCGCGGACCTGTTCTTGGATAAAATGTAGCTATCAACTACATAGCTTTTAAGGGGAGTAGCTAATTACAATAAAGTCGTCATGACAGGGATCTCCCATAAATCCCTCGGCTTTATTGGCAACGGATATACGGTTGTTAGCAAGACCTTTACCAAATGGGGTAAAGGTCTTTTCCTATGCTTTTTTAGACCTTTGCCTATGGGGCAAAGGTCTTTTTCGTAGGAAAGCAGCTTTACAGAAAAAGAACTAAAGGGAGTGGGCGTCATGGAATT | DNA template used for transcription termination assays of *ykoY* RS |
| Bha_ykoY_ RS_size_ marker_ DNA_ template | TAAAAAATGGTTGACCGCGGACCTGTTCTTGGATAAAATGTAGCTATCAACTACATAGCTTTTAAGGGGAGTAGCTAATTACAATAAAGTCGTCATGACAGGGATCTCCCATAAATCCCTCGGCTTTATTGGCAACGGATATACGGTTGTTAGCAAGACCTTTACCAAATGGGGTAAAGGTCTTTTCCTATGCTTTTTTAGACCTTTGCCTATGGGGCAAAGGTCAAAAACGTAGGAAAGCAGCTTTACAGAAAAAGAACTAAAGGGAGTGGGCGTCATGGAATT | DNA template used for full-length marker for transcription termination assays of *ykoY* RS |
| Bha_ykoY_ RSM1_DNA_ template | TAAAAAATGGTTGACCGCGGACCTGTTCTTGGATAAAATGTAGCTATCAACTACATAGCTTTTAAGGGGAGTAGCTAATTACAATAAAGTCGTCATGACAGGGATCTCCCATAAATCCCTCGGCTTTATTGGCAACGGATATACGGTTGTTAGCAAGACCTTTACCAAATGGGGTAAAGGTCTTTTTCGTAGGAAAGCAGCTTTACAGAAAAAGAACTAAAGGGAGTGGGCGTCATGGAATT | DNA template used for transcription termination assays of *ykoY* RSM1 |
| Bha_ykoY_ RSM1_size_ marker_DNA_ template | TAAAAAATGGTTGACCGCGGACCTGTTCTTGGATAAAATGTAGCTATCAACTACATAGCTTTTAAGGGGAGTAGCTAATTACAATAAAGTCGTCATGACAGGGATCTCCCATAAATCCCTCGGCTTTATTGGCAACGGATATACGGTTGTTAGCAAGACCTTTACCAAATGGGGTAAAGGTCAAAAACGTAGGAAAGCAGCTTTACAGAAAAAGAACTAAAGGGAGTGGGCGTCATGGAATT | DNA template used for full-length marker for transcription termination assays of *ykoY* RSM1 |
| CMF_1 | TAAAAAATGGTTGACCGCGGACC | Forward primer used to amplify all *ykoY* DNA templates |
| CMF_2 | CATGACGCCCACTCCCTTTAGTTC | Reverse primer used to amplify all *ykoY* DNA templates |
